# Biocatalytic Oxidative Cascade for the Conversion of Fatty Acids into α‐Ketoacids via Internal H_2_O_2_ Recycling

**DOI:** 10.1002/anie.201710227

**Published:** 2017-12-06

**Authors:** Somayyeh Gandomkar, Alexander Dennig, Andela Dordic, Lucas Hammerer, Mathias Pickl, Thomas Haas, Mélanie Hall, Kurt Faber

**Affiliations:** ^1^ Department of Chemistry University of Graz Heinrichstrasse 28 8010 Graz Austria; ^2^ Austrian Center of Industrial Biotechnology c/o Department of Chemistry University of Graz Heinrichstrasse 28 8010 Graz Austria; ^3^ Creavis Evonik Industries, Bau 1420 Paul Baumann Strasse 1 45772 Marl Germany

**Keywords:** 2-hydroxyacid oxidase, bio-based chemicals, biocatalysis, H_2_O_2_ recycling, P450

## Abstract

The functionalization of bio‐based chemicals is essential to allow valorization of natural carbon sources. An atom‐efficient biocatalytic oxidative cascade was developed for the conversion of saturated fatty acids to α‐ketoacids. Employment of P450 monooxygenase in the peroxygenase mode for regioselective α‐hydroxylation of fatty acids combined with enantioselective oxidation by α‐hydroxyacid oxidase(s) resulted in internal recycling of the oxidant H_2_O_2_, thus minimizing degradation of ketoacid product and maximizing biocatalyst lifetime. The O_2_‐dependent cascade relies on catalytic amounts of H_2_O_2_ and releases water as sole by‐product. Octanoic acid was converted under mild conditions in aqueous buffer to 2‐oxooctanoic acid in a simultaneous one‐pot two‐step cascade in up to >99 % conversion without accumulation of hydroxyacid intermediate. Scale‐up allowed isolation of final product in 91 % yield and the cascade was applied to fatty acids of various chain lengths (C6:0 to C10:0).

Fatty acids obtained from renewable resources constitute an abundant pool of homogeneous carbon‐containing compounds, which upon functionalization can be incorporated in further synthetic transformations towards bio‐based chemicals.^1^ The use of biocatalytic tools combined with strategies to minimize the dependence on stoichiometric cofactors and/or reagents overall contributes to improving the sustainability of such processes: renewable resources as starting materials, mild reaction conditions, and high atom‐efficiency. Cytochrome P450 enzymes^2^ are effective in the oxyfunctionalization of C−H bonds in fatty acids with terminal to α‐regioselectivity.^3^ Their practical applicability is, however, impaired by the dependence on complex electron transport chains to mediate reductive oxygen activation by the heme prosthetic group.^4^ A few P450s known to act as peroxygenases (CYP152 family)^5^ utilize H_2_O_2_ as a shortcut to generate the reactive oxoferryl species compound I via the so‐called peroxide shunt pathway,^6^ and include P450_SPα_,3b P450_BSβ_,^7^ P450_CLA_,^8^ and OleT_JE_.^9^ Due to the generally poor stability of proteins (in particular the heme group itself) towards H_2_O_2_,^8^, ^10^ stoichiometric use of this oxidant in enzymatic oxidative processes is not desired. In situ H_2_O_2_ generation protocols relying on the reduction of oxygen exist and can be cathodic^11^ or mediated by the coupled enzymatic^12^ or flavin‐mediated light‐driven^13^ oxidation of a sacrificial electron donor. To the best of our knowledge, no enzymatic internal H_2_O_2_ recycling protocol has been described, in which H_2_O_2_ is consumed and regenerated within a single enzymatic cascade that does not rely on a sacrificial co‐substrate.

The functionalization of fatty acids to α‐ketoacids is attractive from a synthetic standpoint as follow‐up chemistry can grant access to a multitude of renewable‐based synthons: amino acids, aldehydes, amines, C1‐truncated (odd‐numbered) carboxylic acids, and even alkanes.1b To overcome the abovementioned shortcomings of H_2_O_2_‐mediated enzymatic oxidation protocols, we envisioned a one‐pot two‐step enzymatic cascade^14^ for the conversion of saturated fatty acids to α‐ketoacids. Regioselective α‐hydroxylation by P450 monooxygenase in the peroxygenase mode was combined with enantioselective oxidation of the hydroxyacid intermediate by α‐hydroxyacid oxidase. In theory, only catalytic amounts of peroxide are necessary to kick‐start the reaction, which overall relies on the four‐electron reduction of oxygen, releasing water as sole by‐product (Scheme 1).

**Scheme 1:**
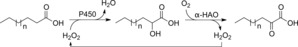
Enzymatic air oxidation of fatty acids to α‐ketoacids via internal H_2_O_2_ recycling in a one‐pot two‐step cascade. P450: P450 monooxygenase in peroxygenase mode; α‐HAO: α‐hydroxyacid oxidase.

For the first oxidative step, P450 from *Clostridium acetobutylicum* (P450_CLA_) was selected owing to its reported activity in the regioselective α‐hydroxylation of fatty acids.^8^ P450_CLA_ was prepared according to a published procedure^15^ and used in purified form for the hydroxylation of octanoic acid (**1**) in the presence of increasing H_2_O_2_ concentration. Regioselective α‐hydroxylation was confirmed and 2‐hydroxyoctanoic acid (**2**) was detected as the sole newly formed product. The reaction was particularly efficient when substoichiometric amounts of peroxide (0.03–0.1 equiv) were used, as monitored by (up to) full incorporation of oxygen (ratio [**2**]/[H_2_O_2_]≈1, Figure 1). Increasing the oxidant concentration expectedly resulted in enhanced conversion, concomitant with a decrease in the [**2**]/[H_2_O_2_] ratio, indicating less efficient hydroxylation, due to partial decomposition of peroxide^16^ and/or catalyst inactivation.^8^ P450_CLA_ enantioselectivity was independent of conversion levels and (*S*)‐**2** was furnished consistently with ca. 36 % *ee*. This set a key requirement for the successful quantitative conversion of **1** into **3** in a cascade reaction (Scheme 1), as formation of the intermediate product **2** with moderate *ee* values necessitates the employment of two stereocomplementary oxidases in the second step of the cascade. To that end, we selected (*S*)‐specific α‐hydroxyacid oxidase from *Aerococcus viridans* [(*S*)‐α‐HAO]^17^ and a recently identified d‐lactate oxidase from *Gluconobacter oxydans* (GO‐LOX)^18^ reported to be (*R*)‐specific for several short‐chain 2‐hydroxyacids [(*R*)‐α‐HAO]. Both α‐HAOs were tested for activity on *rac*‐**2** in the presence of a catalase and were found to be active and highly enantioselective. Both (*R*)‐ and (*S*)‐**2** were obtained in enantiomerically pure form upon action of (*S*)‐α‐HAO and GO‐LOX, respectively (Schemes S1 and S2 and Figure S5).

**Figure 1:**
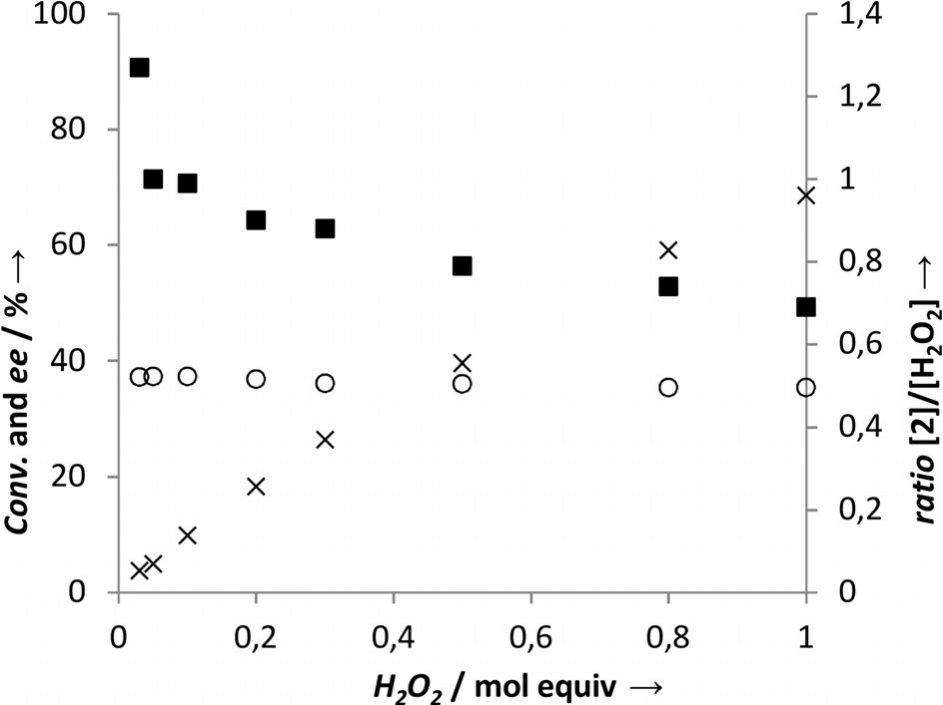
Oxidation of octanoic acid (**1**) to 2‐hydroxyoctanoic acid (**2**) by P450_CLA_. Reaction conditions: 5 μm P450_CLA_, 10 mm **1**, H_2_O_2_ as indicated, Pi buffer (pH 7.4, 100 mm), 10 % EtOH (cosolvent), RT, 24 h, 170 rpm. (×) conversion; (○) *ee* of (*S*)‐**2**; (▪) ratio [**2**]/[H_2_O_2_].

The cascade combining the two oxidation steps in a simultaneous one‐pot fashion was first tested with P450_CLA_ and (*S*)‐α‐HAO with substoichiometric amounts of H_2_O_2_. Analysis of the conversion of **1** revealed formation of final oxo product **3** along with accumulation of intermediate product **2**. Importantly, the data confirmed that internal recycling of H_2_O_2_ is possible and conversion to **3** is only limited by the poor enantioselectivity of P450_CLA_ (max. 36 % *ee* towards (*S*)‐**2**, that is, 68:32 ratio of (*S*)/(*R*)‐enantiomers), furnishing max. 68 % yield of **3** and 32 % yield of **2** using 0.32 equiv of H_2_O_2_ (Table S3). Notably, the amount of accumulated (*R*)‐**2** accurately matched the amount of peroxide used, highlighting that formation of (*R*)‐**2** is a dead‐end pathway when the two enzymes are employed.

The addition of (*R*)‐α‐HAO to prevent accumulation of (*R*)‐**2** resulted in a three‐enzyme one‐pot cascade, which was first tested with 0.1 equiv of H_2_O_2_ (1 mm) and 10 mm octanoic acid. The adjustment of all three enzyme concentrations was crucial to avoid accumulation of 2‐hydroxyacid by maintaining a constant supply of H_2_O_2_ (i.e. fast second oxidation step), while the first step should be rapid enough to avoid decomposition^16^ of H_2_O_2_. An additional issue is the sensitivity of 2‐oxoacids to (non‐enzymatic) H_2_O_2_‐mediated decarboxylation, which furnishes C1‐truncated fatty acids and requires keeping the H_2_O_2_ concentration low (Table S1). Initial trials performed with 5–10 μm P450_CLA_ combined with 0.5 mg mL^−1^ (*S*)‐α‐HAO (≈12 μm) and 2.0 mg mL^−1^ GO‐LOX (≈31 μm) led to 80–89 % conversion of **1** to **3** within 24 h without detectable amount of **2**. This corresponds to roughly 8 full cycles of the peroxide and a proof‐of‐principle for the internal enzymatic recycling of H_2_O_2_ (Table S4). Further tests focused on improving both the H_2_O_2_ turnover number and the extent of conversion by varying oxidant and enzyme concentrations. Under optimized conditions, 77 % and 98 % conversion levels could be obtained using 0.1 and 0.3 equiv of H_2_O_2_, respectively (Table 1, entries 4 and 5, and Tables S5 and S6). Thus, the combination of three enzymes in tandem allowed internal recycling of the peroxide, leading to close to quantitative conversion of the fatty acid. The recycling was more efficient at low peroxide concentration (higher TON${}_{H{}_{2}O{}_{2}}$ , Table 1, entries 1–3).

**Table 1:** Enzymatic oxidation of **1** to **3** via internal H_2_O_2_ recycling performed in a three‐enzyme (A) or two‐enzyme (B) one‐pot cascade. 
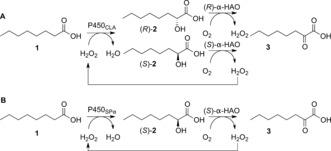

	Cascade	A. Three enzymes^[a]^		B. Two enzymes^[b]^	
Entry	H_2_O_2_ [equiv]	Conv. [%]	TON${}_{H{}_{2}O{}_{2}}$	Conv. [%]	TON${}_{H{}_{2}O{}_{2}}$
1	0.01	20	20.0	n.d.	n.d.
2	0.02	30	15.0	14	7.0
3	0.5	57	11.4	35	7.0
4	0.1	77	7.7	66	6.6
5	0.3	98	3.3	99	3.3

Reactions conditions: [a] 10 mm **1**, H_2_O_2_ as indicated, 5 μm P450_CLA_, 0.1 mm FMN, 12 μm (*S*)‐α‐HAO, 3 μm GO‐LOX, Pi buffer (pH 7.4, 100 mm), 10 % EtOH (cosolvent), RT, 24 h, 170 rpm. [b] 10 mm **1**, H_2_O_2_ as indicated, 5 μm P450_SPα_, 0.1 mm FMN, 24 μm (*S*)‐α‐HAO, Pi buffer (pH 7.4, 100 mm), 10 % EtOH (cosolvent), RT, 24 h, 170 rpm. n.d. not determined. Conversion [%] to final product (yield).

Initiation of the cascade by an enantioselective α‐hydroxylation yielding enantiopure 2‐hydroxyacid implies that only a single oxidase is necessary in the second oxidative step. In an attempt to simplify the system, P450_SPα_ was selected in place of P450_CLA_. The conversion of **1** was confirmed to proceed with high enantioselectivity^19^ towards formation of (*S*)‐**2** (>99 % *ee*, Table S2 and Figure S6) with efficiency comparable to that of P450_CLA_ (Figure 1). The two‐enzyme one‐pot cascade was tested at various concentrations of H_2_O_2_, and full conversion could be obtained using 0.3 equiv of peroxide (i.e. 0.01 % solution) after 24 h reaction time (Table 1, entry 5). Detailed analysis of the reaction progression at lower peroxide concentrations (Figure 2) revealed that the reaction, although incomplete, ceased after a few hours (max. 66 % conversion after 4 h using 0.1 equiv H_2_O_2_), which was attributed to the spontaneous disproportionation of H_2_O_2_,^16^ thereby depleting the reaction mixture of oxidant.

**Figure 2:**
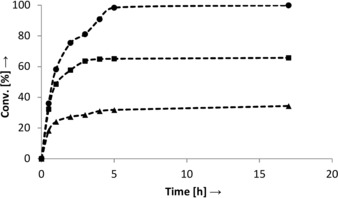
Time profile of the conversion of **1** to **3** in the two‐enzyme cascade setup (B) at various H_2_O_2_ concentrations: (▴) 0.05 equiv, (▪) 0.1 equiv, (•) 0.3 equiv (only traces of **2** detected). Reaction conditions: 10 mm **1**, 5 μm P450_SPα_, 0.1 mm FMN, 24 μm (*S*)‐α‐HAO, Pi buffer (pH 7.4, 100 mm), 10 % EtOH (cosolvent), RT, 170 rpm.

A second portion of peroxide was added in both cases after 5 h (0.5 and 1 mm H_2_O_2_, Table 2). Comparison of conversion levels obtained after single and double addition of peroxide clearly showed that both enzymes were still active after 5 h and more importantly, the H_2_O_2_ supply was indeed limited since in both cases (0.5 and 1 mm), a significant increase in conversion was observed after the second addition of peroxide (Table 2, entries 1, 2 vs. 3, 4). Also, comparison of peroxide turnover numbers reveals that at comparable total H_2_O_2_ supply (1 mm in total), the recycling is more efficient in the double addition mode, with ≈25 % more product formed (Table 2, entries 2 and 3). Similarly, double peroxide addition boosted conversion levels tested in the three‐enzyme cascade (Table 2) and led to more efficient oxidation at a comparable final H_2_O_2_ concentration. Collectively, the data suggest that H_2_O_2_ decomposition is the major factor limiting the efficiency of the internal recycling at low peroxide concentration.

**Table 2:** Single vs. double addition of H_2_O_2_ in the conversion of **1** to **3** in the three‐enzyme (A) or two‐enzyme (B) cascade setup (24 h reaction time).

	Cascade	A. Three enzymes^[b]^		B. Two enzymes^[c]^	
Entry	H_2_O_2_ [mm]	Conv. [%]^[b]^	TON${}_{H{}_{2}O{}_{2}}$	Conv. [%]^[b]^	TON${}_{H{}_{2}O{}_{2}}$
1	0.5	60	12.0	33	6.6
2	0.5+0.5^[a]^	92	9.2	76	7.6
3	1	81	8.1	61	6.1
4	1 + 1^[a]^	99	5.0	95	4.8

[a] Second H_2_O_2_ portion added after 5 h. Reaction conditions: [b] 10 mm **1**, H_2_O_2_ as indicated, 5 μm P450_CLA_, 0.1 mm FMN, 24 μm (*S*)‐α‐HAO, 15 μm GO‐LOX, Pi buffer (pH 7.4, 100 mm), 10 % EtOH (cosolvent), RT, 170 rpm. [c] H_2_O_2_ as indicated, 5 μm P450_SPα_, 0.1 mm FMN, 24 μm (*S*)‐α‐HAO, Pi buffer (pH 7.4, 100 mm), 10 % EtOH (cosolvent), RT, 170 rpm. Conversion [%] to final product (yield).

Finally, both oxidative cascades were implemented at higher substrate concentrations, aiming at increasing the productivity of the system (Table 3). The peroxide concentration was set to 1.0 mm in all cases to allow comparison of the recycling efficiency at varying substrate concentrations. Internal recycling of H_2_O_2_ was most efficient on 20 mm substrate in the three‐enzyme cascade (Table 3, entry 2), furnishing highest amount of product **3** (10.6 mm, 1.7 g L^−1^). In both cascades, conversion levels decreased as substrate concentration increased. Importantly, while inhibition can be confidently expected in the three‐enzyme cascade at 50 mm of **1** (no formation of final product **3** observed along with a small amount of intermediate **2** detected), the two‐enzyme system (cascade B) seems more tolerant to elevated fatty acid concentrations, as improved conversion levels compared to cascade A starting at 30 mm substrate concentration could be reached with no accumulation of intermediate product **2**.

**Table 3:** Conversion of **1** to **3** at various substrate concentrations in presence of 1.0 mm H_2_O_2_ in both oxidative cascade setups.

	Cascade	A. Three enzymes^[a]^		B. Two enzymes^[b]^	
Entry	[**1**] [mm]	Conv. [%]	TON${}_{H{}_{2}O{}_{2}}$ ^[c]^	Conv. [%]	TON${}_{H{}_{2}O{}_{2}}$ ^[c]^
1	10	79	7.9	66	6.6
2	20	53	10.6	38	7.6
3	30	25	7.5	30	8.9
4	40	10	4.1	20	8.0
5	50	3^[d]^	n.a.	11	5.7

Reaction conditions: [a] **1** as indicated, 10 μm P450_CLA_, 0.1 mm FMN, 12 μm (*S*)‐α‐HAO, 15 μm GO‐LOX, Pi buffer (pH 7.4, 100 mm), 10 % EtOH (cosolvent), RT, 24 h, 170 rpm. [b] **1** as indicated, 5 μm P450_SPα_, 0.1 mm FMN, 24 μm (*S*)‐α‐HAO, Pi buffer (pH 7.4, 100 mm), 10 % EtOH (cosolvent), RT, 24 h, 170 rpm. [c] Corresponds to the amount of **3** formed (mm). [d] Hydroxy product **2** as final product. n.a. not applicable. Conversion [%] to final product (yield).

The reaction was conducted on a semi‐preparative scale and the following results were obtained. After 17 h, cascade A (60 mL) led to 98 % conversion of **1** resulting in 74 % yield (70 mg of **3** isolated with 90 % purity) and cascade B (35 mL) allowed isolation of 50 mg of **3** at a 97 % conversion level and 91 % yield (92 % purity, see the Supporting Information and Figures S13–S15). Finally, fatty acids of varying chain lengths (C6:0, C7:0, and C10:0) were tested in both setups and could be successfully transformed to their 2‐oxoacids. Decanoic acid was most reactive and was transformed in up to 93 % conversion to 2‐oxodecanoic acid in a 0.01 % peroxide solution (Table S8).

The internal recycling of H_2_O_2_ could be successfully implemented in a three‐ and two‐enzyme one‐pot cascade for the conversion of saturated fatty acids to α‐ketoacids. The system displays attractive features, notably the use of peroxide as a “catalytic” reagent in substoichiometric amounts, as well as high‐atom efficiency and sustainable character of the reaction, producing water as the sole by‐product and using air as the major oxidant. With this setup, the use of P450 monooxygenases in the peroxygenase mode at low H_2_O_2_ concentrations (≤0.01 %) appears attractive for synthetic applications, owing to improved enzyme lifetime. Additionally, non‐enzymatic oxidative decarboxylation of 2‐oxoacid products is completely avoided and no reaction engineering techniques (e.g. in situ product removal) are required. Altogether, the biocatalytic oxidative cascade combining P450s in the peroxygenase mode with α‐hydroxyacid oxidase(s) is a promising tool for the functionalization of bio‐based chemicals and contributes to more sustainable synthetic routes.

## Experimental Section

Reactions were performed in duplicate in closed glass vials in 1 mL buffer (KPi, 100 mm, pH 7.4), as described in the text. Derivatization for GC and GC‐MS analysis was performed by silylation of the organic phase (Figure S8–S12). Protocols for production of all biocatalysts, reaction conditions, and analytical methods can be found in the Supporting Information. P450_SPα_ was produced using pDB‐HisGST vector obtained from the DNASU plasmid repository (Berkeley Structural Genomics Center).^20^

## Conflict of interest

The authors declare no conflict of interest.

## Supporting information

As a service to our authors and readers, this journal provides supporting information supplied by the authors. Such materials are peer reviewed and may be re‐organized for online delivery, but are not copy‐edited or typeset. Technical support issues arising from supporting information (other than missing files) should be addressed to the authors.

Supplementary
