# Supplementary material for: Biocatalytic Oxidative Cascade for the Conversion of Fatty Acids into α‐Ketoacids via Internal H2O2 Recycling
Source: Angew Chem Int Ed Engl. 2017 Dec 6;57(2):427–30. doi: 10.1002/anie.201710227 (PMC5768024; doi:10.1002/anie.201710227)
Supplement: Supplementary file 1 — Supplementary [file ANIE-57-427-s001.pdf]

Angewandte Chemie
Eine Zeitschrift der Gesellschaft Deutscher Chemiker

Supporting Information

Biocatalytic Oxidative Cascade for the Conversion of Fatty Acids to α-Ketoacids via Internal H_2O_2 Recycling

Somayyeh Gandomkar, Alexander Dennig, Andela Dordic, Lucas Hammerer, Mathias Pickl, Thomas Haas, Mélanie Hall, and Kurt Faber**

anie_201710227_sm_miscellaneous_information.pdf

Supporting Information Table of Contents

1. Material and methods section......2

1.1. Enzyme and chemicals 2

1.2. Biocatalysts......2

1.2.1. Cloning, expression and purification of $P450_{CLA}$ 2

1.2.2. Expression and purification of GO-LOX [(*R*)-α-HAO] 3

1.2.3. Cloning, expression and purification of (*S*)-α-HAO A95G from *Aerococcus viridans* 4

1.2.4. Cloning, expression and purification $P450_{SP\alpha}$ from *Sphingomonas paucimobilis*......5

1.3. Activity assay of purified enzymes......8

1.3.1. GO-LOX and (*S*)-α-HAO......8

1.3.2. Purified $P450_{SP\alpha}$......9

1.4. Cascade-design......9

1.4.1 General procedure for the three-enzyme cascade setup A......9

1.4.2. General procedure for the two-enzyme cascade setup B......10

1.4.3. General procedure for extraction and derivatization on GC-GC/MS 10

2. Results and Discussion 10

2.1. Stability of 2-oxooctanoic acid (**3**) in presence of hydrogen peroxide......10

2.2. Activity assay for the purified enzymes 11

2.2.1. GO-LOX and (*S*)-α-HAO......11

2.2.2. $P450_{SP\alpha}$ (GST-tagged and cleaved tag preparations)......13

2.3. Testing the cascades 14

2.3.1. Conversion of octanoic acid (**1**) to 2-oxo octanoic acid (**3**) with $P450_{CLA}$/(*S*)-α-HAO......14

2.3.2. Testing cascade A by using $P450_{CLA}$/GO-LOX/(*S*)-α-HAO......16

2.3.2.1. Testing different concentrations of $P450_{CLA}$ 16

2.3.2.2. Testing differents concentration of GO-LOX......16

2.3.2.3. Testing cascade A at different temperatures in presence of 0.2 or 0.5 mM H_2O_2 ..17

2.3.3. Testing cascade B by using $P450_{SP\alpha}$/(*S*)-α-HAO......17

2.4. Scale-up experiments......18

2.4.1. Cascade A......18

2.4.2. Cascade B 18

2.5. Conversion of C6:0, C7:0 and C10:0 fatty acids in cascades A and B......18

3. Analytical methods......19

3.1. GC, GC-MS and NMR......19

3.2. Determination of *ee* value of 2-hydroxyoctanoic acid on chiral GC......20

3.3. GC/ GC-MS chromatograms and NMRs......20

4. References 31

1. Material and methods

1.1. Enzymes and chemicals

All chemicals, catalase (from bovine liver) and lysozyme (from chicken egg) were obtained from Sigma Aldrich (Steinheim, Germany) unless otherwise stated.

The plasmid for expression of GO-LOX enzyme was a kind gift from Prof. Ping Xu and Dr. Chao Gao (Shandong University, China). The pDB-HisGST plasmid used for cloning of $P450_{SP\alpha}$ was obtained from the DNASU plasmid repository.[1]

1.2. Biocatalysts

1.2.1. Cloning, expression and purification of $P450_{CLA}$ (in-house number pEG306)

Cloning of $P450_{CLA}$ from *Clostridium acetobutylicum*[2] was performed as reported elsewhere.[3]

For heterologous expression, cells were grown overnight (140 rpm, 37 °C) in a 50 mL shake flask as pre-culture in 10 mL lysogeny broth (LB) medium supplemented with 50 µg/mL kanamycin. For preparing the main culture, 1 mL of grown pre-culture was transferred into 100 mL TB medium pre-filled into a 1 L shake flask supplemented with 50 µg/mL kanamycin and 100 µL of a sterile filtered trace elements solution[4] and incubated at 140 rpm and 37 °C. At an optical density (OD_{600}) of 0.8, the expression was induced by adding 100 µL of 100 mM IPTG solution (isopropyl *β*-D-1-thiogalactopyranoside) and 200 µL of 0.5 M *δ*-aminolevulinic acid (ALA) solution. After incubation for 20 h at 25 °C, the cells were centrifuged for 15 min at 3,000 x g and 4 °C. The supernatant was discarded and the pellet was frozen at -20 °C. Frozen cell pellets were resuspended in 20 mL purification buffer A (KPi, 100 mM, pH 7.0, 20% glycerol, 300 mM KCl and 50 mM imidazole). Lysozyme was added (1 mg/mL) followed by incubation at 37 °C for 2 h. Cells were finally disrupted by sonication (1 min at 30% amplitude; 2 sec on, 4 sec off, Digital Cell Disrupter, Branson, Emerson Electric). Cell debris was removed by ultracentrifugation (23,500 x g, 20 min, 4 °C). The cell free lysates were pressed through a sterile 0.45 µm Rotilabo® syringe filter (Roth, Karlsruhe, Germany) to eliminate residual particles.

Filtered cell free lysates were purified by a 5 mL His-Trap™ FF column (GE Healthcare Europe GmbH), which was washed with 50 mL water. For eluting the undesired enzymes, the column was washed with 50 mL (10 column volumes) of buffer A (without glycerol). After the washing step, $P450_{CLA}$ was eluted by buffer B (KPi, 100 mM, pH 7.0, 300 mM KCl and 400 mM imidazole) and the fraction containing $P450_{CLA}$ (~20 to 30 mL) was dialyzed for 36 h with imidazole-free buffer C (KPi, 100 mM, pH 7.0, 300 mM KCl) to remove residual imidazole using a dialysis tubing cellulose membrane (14 kDa cutoff, Sigma Aldrich, Steinheim, Germany). The purified protein was dialyzed three times against 300 mL buffer C (3 x 12 h) at 4 °C under continuous and slow stirring (50 rpm). Both activity and concentration of the enzyme were measured via analysis of reduced CO difference spectra as published by Omura and Sato.[5] Upon addition of 300 mM KCl, no visible precipitation occurred during the entire dialysis procedure and active P450 concentrations between 10 to 20 µM were regularly obtained without further concentration steps.

1.2.2. Expression and purification of GO-LOX [(*R*)-α-HAO] (in-house number pEG359)

The plasmid for expression of GO-LOX enzyme from *Gluconobacter oxydans* 621H was a kind gift from Prof. Ping Xu and Dr. Chao Gao (Shandong University, China).[6]

The plasmid was transformed into *E. coli* BL21 (DE3) and one colony was used to grow a new pre-culture in 10 mL LB medium containing 100 µg/mL ampicillin. For preparing the main culture, 1 mL of grown pre-culture was transferred into 100 mL LB medium pre-filled into a 1 L shake flask supplemented with 100 µg/mL ampicillin solution and incubated at 140 rpm and 37 °C. At an optical density (OD_{600}) of 0.4–0.6, the expression was induced by adding 1 mM IPTG solution and incubation was carried out at 16 °C for further 10 h. Cells were harvested, washed and disrupted by sonication (amplitude 20%, 6 sec on, 6 sec off, total time 15 min) in binding buffer (pH 7.4, 20 mM sodium phosphate, 500 mM NaCl and 20 mM imidazole) supplemented with 1 mM phenylmethylsulfonyl fluoride (PMSF) and 10% glycerol.

After centrifugation (15 min at 23,500 x g and 4 °C), the resulting supernatant was loaded onto a HisTrap HP column (5 mL) equilibrated with binding buffer (pH 7.4, 20 mM sodium phosphate, 500 mM NaCl and 20 mM imidazole), and eluted with elution buffer (20 mM NaPi, pH 7.4, 500 mM NaCl and 500 mM imidazole) to obtain the purified target enzyme (Figure S1). The purified enzyme was finally concentrated by ultrafiltration in VIVASPIN tubes (MWCO 10 kDa, 4,688 x g at 4 °C) and stored in 50 mM Tris-HCl buffer (pH 7.4).

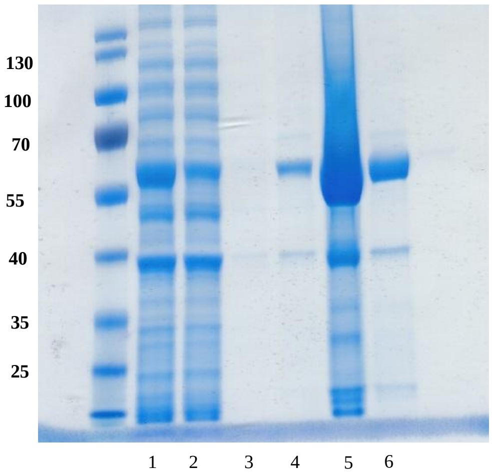

Figure S1. 10% SDS-PAGE during GO-LOX purification. Lane 1: supernatant after disruption, Lane 2: flow-through, Lane 3: washing fraction, Lane 4-6: elution fractions. Ladder: PageRuler prestained protein ladder.

1.2.3. Cloning, expression and purification of (*S*)-α-HAO A95G from *Aerococcus viridians* (in-house number pEG358)

(*S*)-α-HAO[7] is a FMN-dependent and (*S*)-specific α-hydroxyacid oxidase from *A. viridans*. The gene coding for the protein bearing mutation A95G was ordered (Thermo Fisher Scientific) and cloned in pET28a(+) using restriction sites *Xho*I and *Nde*I such that the protein bears a His6-tag.

***(S)*-α-HAO A95G from *Aerococcus viridans* nucleotide sequence (ordered gene)**

ATGAACAACAACGACATCGAATATAATGCACCGAGCGAGATCAAATATATCGATGTGGTTAATACCTACGATC

TGGAAGAAGAAGCCAGCAAAGTTGTTCCGCATGGTGGTTTTAACTATATTGCCGGTGCAAGCGGTGATGAATG

GACCAAACGTGCAAATGATCGTGCATGGAAACATAAACTGCTGTATCCGCGTCTGGCACAGGATGTTGAAGCA
CCGGATACCAGCACCGAAATTCTGGGTCATAAAATCAAAGCACCGTTTATTATGGCACCGATTGGTGCACATG
GTCTGGCCCATGCAACCAAAGAAGCAGGCACCGCACGCGCAGTTAGCGAATTTGGCACCATTATGAGCATTAG
CGCATATAGCGGTGCAACCTTTGAAGAAATTAGCGAAGGTCTGAATGGTGGTCCGCGTTGGTTTCAGATTTAT
ATGGCAAAAGATGATCAGCAGAACCGCGATATTCTGGATGAAGCAAAAGGTGATGGTGCAACCGCAATTATTC
TGACCGCAGATAGCACCGTTAGCGGTAATCGTGATCGTGATGTGAAAAACAAATTCGTGTATCCGTTTGGTAT
GCCGATTGTTCAGCGTTATCTGCGTGGCACCGCAGAAGGTATGAGCCTGAATAACATTTATGGTGCCAGCAAA
CAGAAAATCAGTCCGCGTGATATCGAAGAAATTGCAGCACATAGCGGTCTGCCGGTTTTTGTTAAAGGTATTC
AGCATCCGGAAGATGCAGATATGGCAATTAAAGCGGGTGCCAGCGGTATTTGGGTTAGCAATCATGGTGCACG
TCAGCTGTATGAAGCTCCGGGTAGCTTTGATACCCTGCCTGCAATTGCCGAACGTGTTAATAAACGTGTTCCG
ATTGTTTTTGATAGCGGTGTTCGTCGTGGTGAACATGTTGCAAAAGCACTGGCCAGCGGTGCAGATGTTGTTG
CACTGGGTCGTCCGGTTCTGTTTGGTCTGGCGCTGGGTGGTTGGCAGGGTGCCTATAGCGTTCTGGATTATTT
TCAGAAAGATCTGACCCGTGTTATGCAGCTGACCGGTAGCCAGAATGTTGAAGATCTGAAAGGTCTGGACCTG
TTTGATAATCCGTATGGCTATGAGTATTAA

For over-expression, 10 mL of autoclaved TB medium was filled in a 50 mL shake flask supplemented with 50 µg/mL kanamycin solution and 10 µL glycerol stock solution, then incubated overnight at 140 rpm and 37 °C. 1 mL of the pre-culture was transferred into 100 mL TB medium pre-filled into a 1 L shake flask supplemented with 50 µg/mL kanamycin solution incubated at 140 rpm and 37 °C. At an optical density (OD_{600}) of 1, the expression was induced by adding 100 µL of 500 mM IPTG solution. After incubation for 20 h at 30 °C, the cells were centrifuged for 15 min at 3,000 x g and 4 °C. The supernatant was discarded and the pellet was frozen at -20 °C until further use. Frozen cell pellets were re-suspended in 20 mL lysis buffer (KPi, 50 mM, pH 7.5, 50 mM imidazole and 1 mg/mL lysozyme) followed by incubation on ice for 2 h. Before sonication, FMN (10 µM final concentration) was added, then cells were disrupted by sonication (Amplitude 20%, 4 sec on, 4 sec off, total time 5 min). Cell debris was removed by ultracentrifugation (14,000 x g, 20 min, 4 °C). The cell free lysates were pressed through a sterile 0.45 µm syringe filter to eliminate residual particles. Filtered cell free lysates were purified by a 5 mL His-Trap™ FF column (GE Healthcare Europe GmbH), which was washed with 50 mL water. For eluting the undesired enzymes, the column was washed with 50 mL (10 column volumes) binding buffer (KPi, 50 mM, pH 7.5, 20 mM imidazole). After the washing step, the target enzyme was eluted with the elution buffer (KPi, 50 mM, pH 7.5, 400 mM imidazole) and stored in buffer (KPi, 50 mM, pH 7.5, 50 mM KCl) by using a desalting column (PD-10 Desalting Column, GE Healthcare). In order to confirm purity, all samples were loaded on 10 % SDS-PAGE (Figure S2).

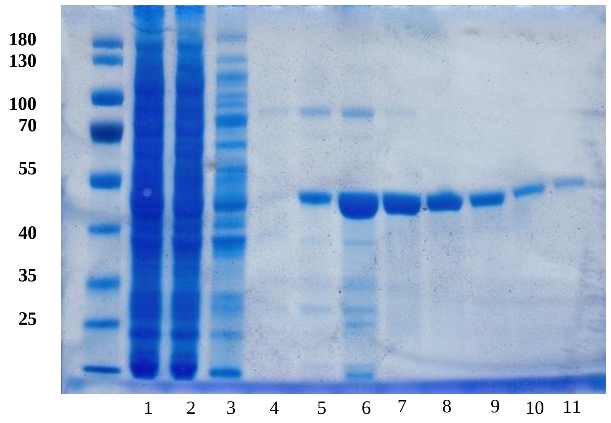

Figure S2. 10% SDS-PAGE monitoring of (*S*)-α-HAO purification. Lane 1: cell free lysate, Lane 2-3: flow through, Lane 4: washing fraction, Lane 5-11: elution fractions. Ladder: PageRuler prestained protein ladder.

Oxidase activity was measured at room temperature using a peroxidase-coupled assay containing 3,5-dichloro-2-hydroxybenzenesulfonic acid (DCHBS) and 4-aminoantipyrine (AAP) as chromogenic substrates (HRP-AAP/DCHBS assay) in combination with lactic acid as substrate.[8] The oxidation of lactic acid by (*S*)-α-HAO releases H_2O_2, which is monitored by the assay.

Assay mixture composition: 200 µL of 0.1 mM AAP and 1 mM DCHBS, 20 µL of 5 mg/mL HRP, 10 µL of 100 mM substrate (*rac*-lactic acid), 680 µL phosphate buffer (100 mM, pH 7). 50 µL cell lysate containing (*S*)-α-HAO was added directly before measuring absorption at 515 nm.

FMN was added to all reaction mixtures to allow full saturation of the enzyme following purification.

1.2.4. Cloning, expression and purification P450$_{SP\alpha}$ from *Sphingomonas paucimobilis* (in-house number pEG371)

The pDB-HisGST vector harboring a GST-tag (fusion protein to enhance soluble expression) followed by a *N*-terminal His$_6$-tag was chosen as expression vector.[1] Additionally, this vector gives the ability to cleave off the GST-tag by a TEV protease. The gene coding for P450$_{SP\alpha}$[9] was ordered from Thermo Fisher Scientific (Germany) and inserted into pDB-HisGST vector using *NdeI* and *XhoI* restriction sites.

P450$_{SP\alpha}$ (CYP152 from *Sphingomonas paucimobilis*) nucleotide sequence (ordered gene)

catATGCCGAAAACACCGCATACCAAAGGTCCGGATGAAACCCTGAGCCTGCTGGCAGATCCGTATCGTTTTA
TTAGCCGTCAGTGTCAGCGTCTGGGTGCAAATGCCTTTGAAAGCCGTTTTCTGCTGAAAAAAACCAATTGTCT
GAAAGGTGCAAAAGCAGCCGAAATCTTTTATGATACCACCCGTTTTGAACGTGAAGGTGCAATGCCGGTTGCA
ATTCAGAAAACCCTGCTGGGTCAGGGTGGTGTTCAGGGTCTGGATGGTGAAACCCATCGTCATCGTAAACAAA
TGTTTATGGGTCTGATGACACCGGAACGTGTTCGTGCACTGGCACAGCTGTTTGAAGCAGAATGGCGTCGTGC
AGTTCCGGGTTGGACCCGTAAAGGTGAAATTGTTTTTTATGATGAACTGCATGAACCGCTGACCCGTGCAGTT
TGTGCATGGGCAGGCGTTCCGCTGCCGGATGATGAAGCAGGTAATCGTGCCGGTGAACTGCGTGCACTGTTTG
ATGCAGCCGGTAGCGCAAGTCCGCGTCATCTGTGGTCACGTCTGGCACGTCGTCGTGTTGATGCATGGGCCAA
ACGTATTATTGAAGGTATTCGTGCAGGTAGCATTGGTAGCGGTAGCGGCACCGCAGCTTATGCAATTGCCTGG
CATCGTGATCGTCATGATGATCTGCTGAGTCCGCATGTTGCAGCAGTTGAACTGGTTAATGTTCTGCGTCCGA

CCGTTGCCATTGCAGTGTATATTACCTTTGTTGCACATGCACTGCAGACCTGTAGCGGTATTCGCGCAGCACT
GGTTCAGCAGCCGGATTATGCAGAACTGTTTGTTCAAGAAGTGCGTCGCTTTTATCCGTTTTTTCCGGCAGTT
GTTGCACGTGCCAGCCAGGATTTTGAATGGGAAGGTATGGCATTTCCGGAAGGTCGTCAGGTTGTTCTGGATC
TGTATGGTAGCAATCATGATGCAGCAACCTGGGCTGATCCGCAAGAATTTCGTCCGGAACGCTTTCGCGCATG
GGATGAAGATAGCTTTAACTTTATTCCGCAGGGTGGCGGTGATCATTATCTGGGTCATCGTTGTCCGGGTGAA
TGGATTGTTCTGGCAATTATGAAAGTTGCAGCACATCTGCTGGTGAATGCAATGCGTTATGATGTTCCGGATC
AGGATCTGAGCATTGATTTTGCACGTCTGCCTGCACTGCCGAAAAGCGGTTTTGTTATGCGTAATGTTCATAT
CGGTGGCTAActcgag

The recombinant plasmid was transformed into *E. coli* NEB5α cell lines for amplification. Transformants were plated on LB agar plates containing kanamycin (50 µg/mL) antibiotic. Colonies were picked for overnight cultures (ONCs) and plasmids were isolated by QIAprep Spin Miniprep Kit. Isolated plasmids were sent for sequencing (all positive, Microsynth) and afterwards transformed into *E. coli* BL21 DE3. ONCs of the transformants were prepared in 50 mL tubes (10 mL LB Medium + 50 µg/mL kanamycin) and incubated at 37 °C and shaken at 120 rpm overnight. ONCs were used to prepare glycerol stocks (500 µL culture + 500 µL 30% glycerol stock) and stored until further use (at -20 °C or -80 °C).

For growing cells, shaking flasks (volume 250 mL or 1 L) were filled with TB medium (100 mL or 330 mL) and autoclaved. After cooling to room temperature, kanamycin (50 µg/mL) and 100 µL of a sterile filtered trace elements solution[4] were added. The prepared medium was inoculated with the ONC (1 mL) and shaken at 37 °C and 120 rpm. When OD_{600} reached 0.6 - 0.8, the cultures were cooled down to room temperature and δ-aminolevulinic acid was added (0.5 mM). Cells were then induced with IPTG (0.1 mM) and the culture was shaken overnight at 20 °C and 120 rpm. The next day the cells were harvested by centrifugation (12,040 x g, 20 min, 4 °C). The cell pellets were washed with buffer (KPi, 100 mM, pH 7.4, 10 mL buffer per g pellet used) and resuspended in the same buffer (if His-tag purification was performed, lysis buffer B was used). For cell disruption, the suspension was ultrasonicated on ice [(30% amplitude, 2 sec on, 4 sec off for 2 min) x 2]. Cell debris was removed by centrifugation (18,800 x g, for 20 min, at 4 °C) and the supernatant (soluble fraction) was used for enzyme purification. The pellet was resuspended in buffer B (KPi, 100 mM, pH 7.5, 100 mM NaCl, 0.8% w/v cholate, 1 mM PMSF and 15% v/v glycerol, 10 mL buffer per g pellet used) and a sample was taken (insoluble fraction). Expression level of the enzyme was analyzed by SDS-PAGE (Figure S3). Both activity and concentration of the enzyme were measured via analysis of reduced CO difference spectra as published.[5]

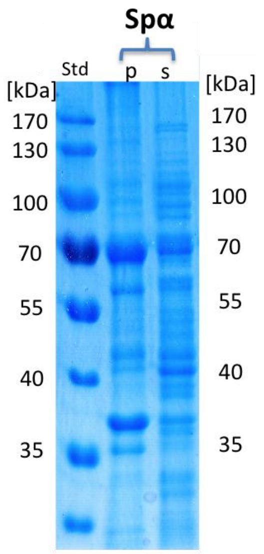

Figure S3. SDS-PAGE results after expression trial of GST-fused SPα using pDB-HisGST vector. MW = 75 kDa (Spα around 45 kDa + GST tag around 30 kDa = 75 kDa). p: pellet fraction; s: supernatant fraction; Std: PageRuler prestained protein ladder.

To purify the *N*-terminal His-tagged GST-fused SPα enzyme, a 5 mL His-Trap™ FF column (GE Healthcare Europe GmbH) was used. After harvesting the cells, the pellets obtained from centrifugation (12,040 x g at 4 °C for 20 min) were washed with buffer (KPi, 100 mM, pH 7.4) and resuspended in lysis buffer (KPi, 100 mM, pH 7.5, 100 mM NaCl, 0.8% w/v cholate, 1 mM PMSF, 30 mM imidazole and 15% v/v glycerol, 10 mL lysis buffer per g cell pellet used) and incubated for 2 h. The suspension was ultrasonicated [(30% amplitude, 2 sec on, 4 sec off for 2 min) x 2] and centrifuged again (38,800 x g at 4 °C for 20 min). The column was washed with lysis buffer (50 mL). Then, the sterile filtered sample (0.45 µm Rotilabo® syringe filter) was loaded onto the column and washed again with lysis buffer (50 mL). Enzyme was eluted with elution buffer (KPi, 100 mM, pH 7.5, containing 100 mM NaCl, 0.8% w/v cholate, 1 mM PMSF, 250 mM imidazole and 15% v/v glycerol, up to 25 mL). Eluted fractions were concentrated by centrifugation in VIVASPIN tubes (MWCO 10 kDa, 4,688 x g at 4 °C).

After concentration, samples were desalted (PD-10 Desalting Columns, GE Healthcare) and eluted in buffer (KPi, 100 mM, pH 7.4 containing 15% glycerol) and stored at 4 °C. Glycerol was removed from the enzyme solution before use in biotransformations by using centrifugation in VIVASPIN tubes (MWCO 10 kDa, 4,688 x g at 4 °C) and loading fresh reaction buffer (KPi, 100 mM, pH 7.4). The purified sample was cleaved with TEV protease.[1a] SDS-PAGE results during loading, after purification and after cleavage of the enzyme are shown in Figure S4.

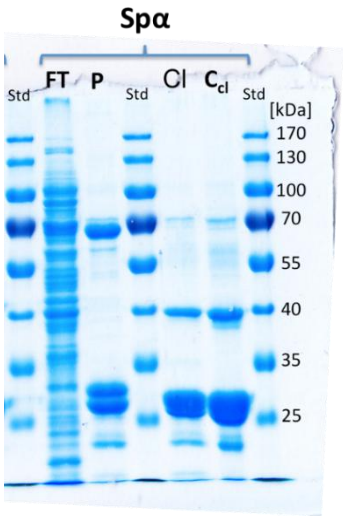

Figure S4. SDS PAGE after protein purification. FT: flowthrough; P: collected eluted fraction; Cl: cleavage fraction (TEV protease added); C_{Cl}: cleavage fraction concentrated; Std: PageRuler prestained protein ladder.

Most of the protein bound to the column and was eluted afterwards (P – pure fraction around 75 kDa). After TEV protease (27 kDa) addition, successful cleavage was monitored (P450$_{SP\alpha}$ at 40 kDa and GST-tag at 25 kDa).

1.3. Activity assay of purified enzymes

1.3.1. GO-LOX and (*S*)-α-HAO

The oxidase activity of both purified hydroxyacid oxidases (GO-LOX and (*S*)-α-HAO) was tested in the oxidation of *rac*-2-hydroxyoctanoic acid (**2**) (Scheme S1-S2). Initial oxidation reactions were performed in 1 mL scale in closed glass vials at 170 rpm for 24 h. Analysis on GC-GC/MS was performed after extraction and derivatization as reported below (1.4.3). Dodecanoic acid (5 mM) was used as internal standard.

In both reactions, ethanol was used as co-solvent. It is a co-solvent of choice for fatty acid-type substrates[3] and is not accepted by the two hydroacid oxidases, which are highly specific for 2-hydroxy acid substrates.

Reaction mixture composition with GO-LOX: 10 mM of *rac*-2-hydroxyoctanoic acid, 10% v/v EtOH, different concentration of GO-LOX (0.5 mg/mL, 1.0 mg/mL and 2.0 mg/mL), 0.05 mg/mL catalase and 1 mL buffer (Tris-HCl, 50 mM, pH 7.4).

Reaction mixture composition with (*S*)-α-HAO: 10 mM of *rac*-2-hydroxyoctanoic acid, 10% v/v EtOH, different concentration of (*S*)-α-HAO (0.5 mg/mL and 1.0 mg/mL), 0.05 mg/mL catalase, 0.1 mM FMN and 1 mL buffer (KPi, 100 mM, pH 7.4).

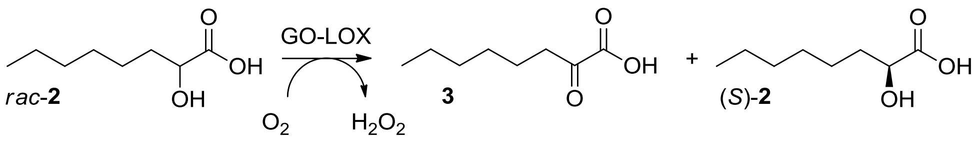

Scheme S1. Oxidation of 10 mM *rac*-2-hydroxyoctanoic acid (**2**) with GO-LOX (catalase implemented to remove H_2O_2)

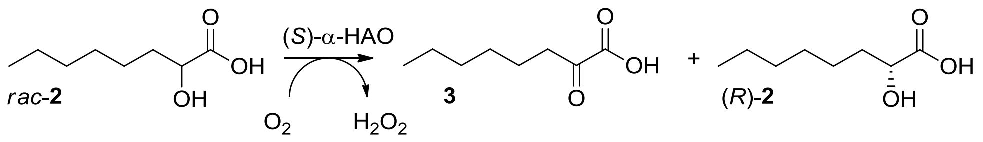

Scheme S2. Oxidation of 10 mM *rac*-2-hydroxyoctanoic acid (**2**) with (*S*)-α-HAO (catalase implemented to remove H_2O_2)

1.3.2. Purified P450$_{SPα}$

Hydroxylation activity of P450$_{SPα}$ was investigated by using the tagged enzyme and the enzyme solution containing the cleaved-tag enzyme (tag still present in solution) in the oxidation of octanoic acid (**1**) (Scheme S3). The oxidation reaction was performed in 1 mL scale in closed glass vials at 170 rpm for 24 h. Analysis on GC-GC/MS was performed after extraction and derivatization as reported below (1.4.3). Dodecanoic acid (5 mM) was used as internal standard.

<u>Reaction mixture composition for single oxidation step</u>: 10 mM of octanoic acid (**1**), 10% v/v EtOH, 2.5 µM tagged or cleaved-tag enzyme solution, 3.0 mM H_2O_2 in 1 mL buffer (KPi, 100 mM, pH 7.4).

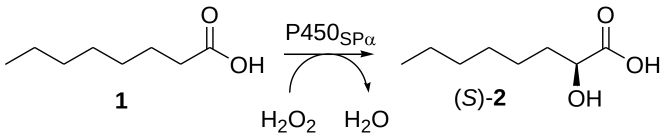

Scheme S3. Oxidation of octanoic acid (**1**) with P450$_{SPα}$

1.4. Cascade-design

1.4.1. General procedure for the three-enzyme cascade setup A

Enzymatic oxidation of octanoic acid (**1**) to 2-oxooctanoic acid (**3**), by employing three enzymes (P450$_{CLA}$, GO-LOX and (*S*)-α-HAO, cascade A) was performed with the following procedure (Scheme S4).

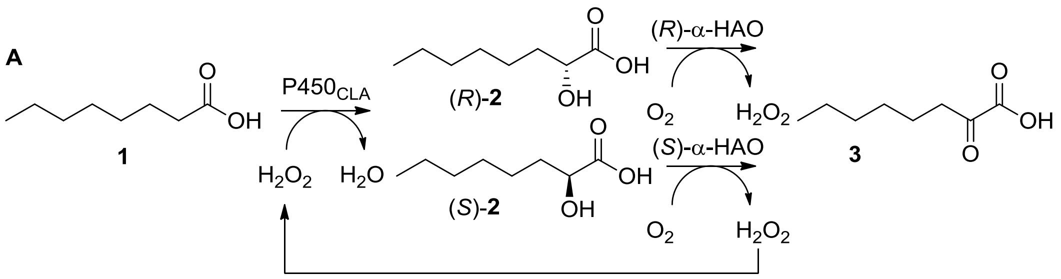

Scheme S4. Conversion of octanoic acid (**1**) to α-ketoacid (**3**) with internal H_2O_2 recycling (**Cascade A**)

The reaction was performed in 1 mL buffer (KPi, 100 mM, pH 7.4) containing 5 µM P450$_{CLA}$, 10 mM octanoic acid, 10 % EtOH (co-solvent), 0.1 mM FMN, (*S*)-α-HAO at various concentrations (0.5 mg/mL and 1.0 mg/mL), GO-LOX at various concentrations (0.2 mg/mL, 0.5 mg/mL, 1.0 mg/mL and 2.0 mg/mL) and H_2O_2 at varying concentrations (0.5 mM to 3 mM). Cascade reactions were performed in duplicates in closed glass-vials at RT and 170 rpm for 24 h.

1.4.2. General procedure for the two-enzyme cascade setup B

Enzymatic oxidation of octanoic acid (**1**) to 2-oxo octanoic acid (**3**), by employing two enzymes ($P450_{SP\alpha}$ and (*S*)-α-HAO, cascade B) was performed with the following procedure (Scheme S5).

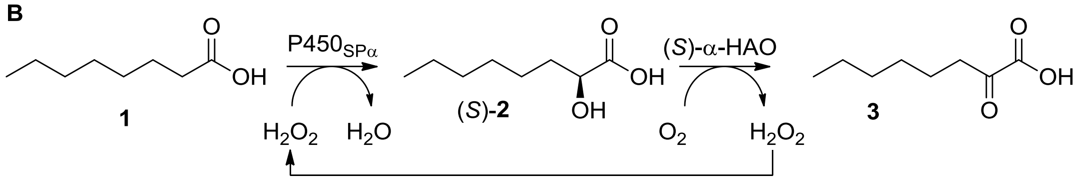

Scheme S5. Conversion of octanoic acid (**1**) to α-ketoacid (**3**) with internal H_2O_2 recycling (**Cascade B**)

The reaction was performed in 1 mL buffer (KPi, 100 mM, pH 7.4), containing 5 µM $P450_{SP\alpha}$ (GST-tagged enzyme), 10 mM octanoic acid, 10 % EtOH (co-solvent), 0.1 mM FMN, 1.0 mg/mL (*S*)-α-HAO and H_2O_2 at varying concentrations (0.5 mM to 3 mM). Cascade reactions were performed in duplicates in closed glass-vials at RT and 170 rpm for 24 h.

1.4.3. General procedure for extraction and derivatization on GC-GC/MS

Analysis on GC-GC/MS was performed after extraction and derivatization. Dodecanoic acid (5 mM) was employed as internal standard. Silylation was found to be the most suitable derivatization method before achiral GC and GC-MS analysis (leading to quantitative yield to corresponding silylated substrate, hydroxy and oxo-products). Silylation of final oxo-product delivered a mixture of derivatized oxo-acid and derivatized enol-form of the oxo-acid.

To this end, 100 µL of 5 M HCl were added to 1 mL aqueous buffer solution (blank or enzymatic reaction mixture or standard) before extraction with EtOAc (2 x 0.5 mL) containing 5 mM dodecanoic acid as internal standard. The combined organic phases were dried over Na_2SO_4. After centrifugation for 1 min, 100 µL were sampled and mixed with 200 µL of 1:1 (BSTFA/TMCS 99:1 - *N,O*-Bistrifluoroacetamide/trimethylsilylchloride)/pyridine solution. After 2 hours at room temperature, samples were directly analyzed on GC-GC/MS.

2. Results and discussion

2.1. Stability of 2-oxooctanoic acid (3) in presence of hydrogen peroxide

Non-enzymatic H_2O_2-mediated oxidative decarboxylation[10] of **3** may reduce the apparent yield of the reaction, thereby producing heptanoic acid as side-product. In order to investigate the H_2O_2-recycling cascade, initial experiments focused on stability of 2-oxooctanoic acid (**3**) in presence of H_2O_2. First, decarboxylation of the ketoacid by H_2O_2 was investigated on a mixture of 10 mM **3** and 5 % EtOH (co-solvent) in buffer (KPi, 100 mM, pH 7.5, 1 mL) in closed glass vials at RT and 170 rpm. H_2O_2 was added at a final concentration of 0.4, 0.8, 1.6 or 3.2 mM. BSA (5µM) was added to a second series of reactions to investigate the possible effect of protein surface catalyzed decarboxylation of 2-ketoacid. 200 µL from each

mixture was taken at four time points (30 min, 1 h, 2 h and 4 h). A calibration curve was prepared using commercially available 2-oxooctanoic acid and GC-MS analytics.

In absence of BSA, decarboxylation of 2-oxooctanoic acid occurred, mostly at higher concentration of peroxide (>3 mM). In presence of BSA, decarboxylation was more pronounced and more advanced at higher concentrations of H_2O_2 (1.6 and 3.2 mM). At 10 mM H_2O_2 decarboxylation was nearly complete (Table S1).

Table S1. Recovered 2-oxooctanoic acid **3** (10 mM) from reaction with H_2O_2 with and without BSA addition, expressed as concentration (mM) calculated from external calibration curve at different time points.

	- BSA						+ BSA					
**Time (h)**	**0**	**0.5**	**1**	**2**	**4**	**24**	**0**	**0.5**	**1**	**2**	**4**	**24**
**[H_2O_2] mM**												
0.4	-	10	10	9.9	9.7	-	-	9.4	8.9	8.0	8.3	-
0.8	-	-	9.8	9.4	9.6	-	-	7.3	7.2	7.2	7.5	-
1.6	-	-	10	10	10	-	-	6.6	6.5	6.0	6.7	-
3.2	8.1	-	-	-	7.0	7.1	7.2	-	-	-	6.3	6.2
10	1.5	-	-	-	0	0	3.4	-	-	-	0	0
0	9.8	-	-	-	9.8	9.8	-	-	-	-	-	-

2.2. Activity assay for purified enzymes

2.2.1. GO-LOX and (*S*)-α-HAO

The oxidase activity of purified hydroxyacid oxidases (GO-LOX and (*S*)-α-HAO) was tested in the oxidation of *rac*-2-hydroxyoctanoic acid (**2**) (Scheme S1-S2). Conversion of 2-hydroxyoctanoic acid with both hydroxyacid oxidases revealed formation of a new product. Chiral GC measurements confirmed the exquisite selectivity of GO-LOX and (*S*)-α-HAO for the (*R*)- and the (*S*)-enantiomer respectively (Figure S5). Detailed methods are provided in section 3.

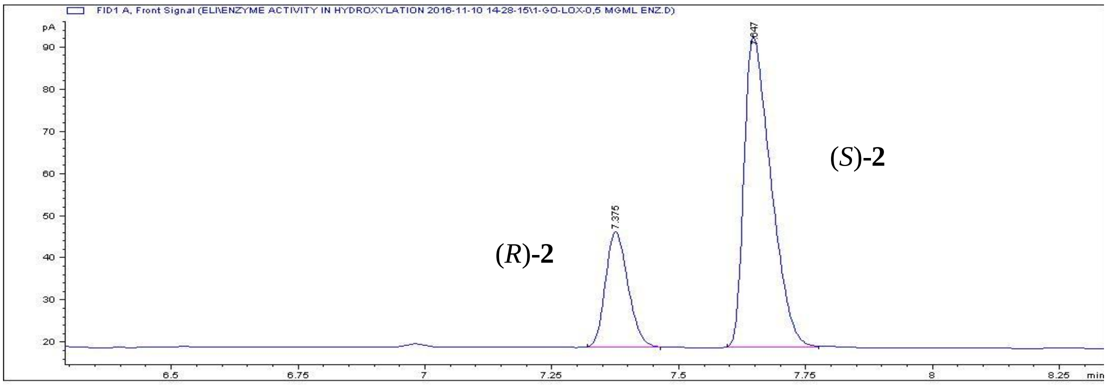

a)

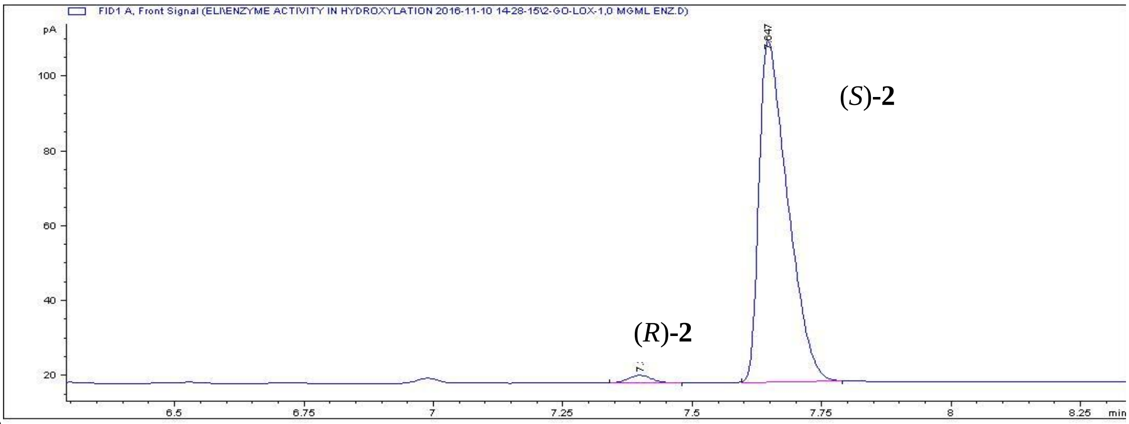

b)

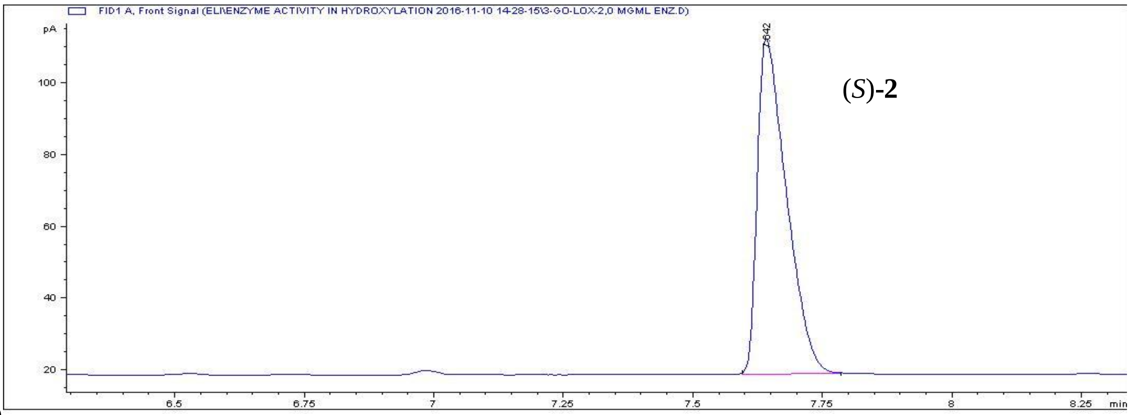

c)

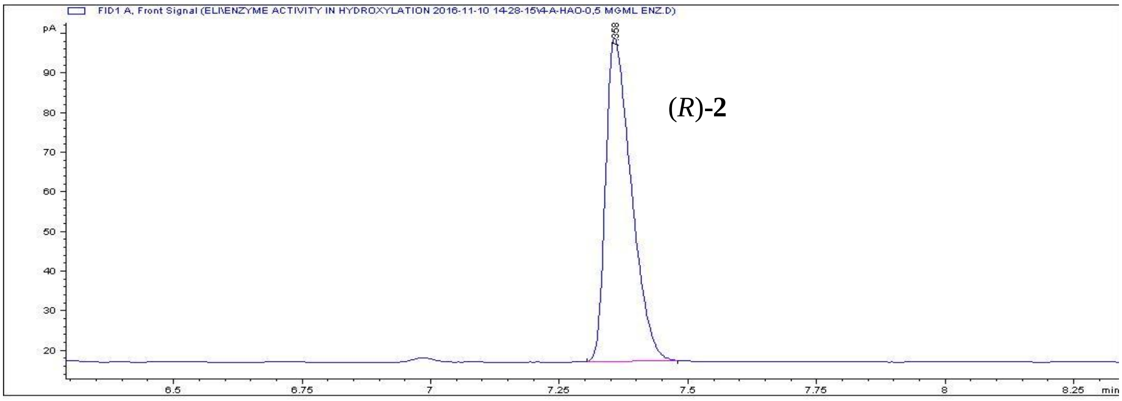

d)

Figure S5. Chiral GC traces obtained from oxidation of 10 mM *rac*-**2** with a) GO-LOX (0.5 mg/mL), b) GO-LOX (1.0 mg/mL), c) GO-LOX (2.0 mg/mL), d) (*S*)-α-HAO (0.5 mg/mL).

2.2.2. P450$_{SP\alpha}$ (GST-tagged and cleaved-tag preparations)

Hydroxylation ability of purified P450$_{SP\alpha}$ (the tagged enzyme and the enzyme preparation containing the free protein with the cleaved tag in solution) was tested on 10 mM **1** in presence of 3.0 mM H_2O_2. Results are shown in Table S2 and Figure S6. Both enzyme preparations were tested in the cascade set-up B in combination with (*S*)-α-HAO (Figure S7). Detailed methods are provided in section 3.

Table S2. Hydroxylation of octanoic acid (**1**) to 2-hydroxyoctanoic acid (**2**) using P450$_{SP\alpha}$ in the presence of 3.0 mM H_2O_2 (analyzed on achiral GC) overnight

	[1] (mM)	[2] (mM)	Conversion %	% of Theoretical yield	Recovery %	Ratio [2]/ [H_2O_2]
Control (no enzyme)	10.2	0	0	-	101.8	-
P450$_{SP\alpha}$ without tag	8.2	2.0	20.0	66.7	102.1	0.7
P450$_{SP\alpha}$ with GST-tag	7.8	2.3	22.9	76.3	101.2	0.8

a) 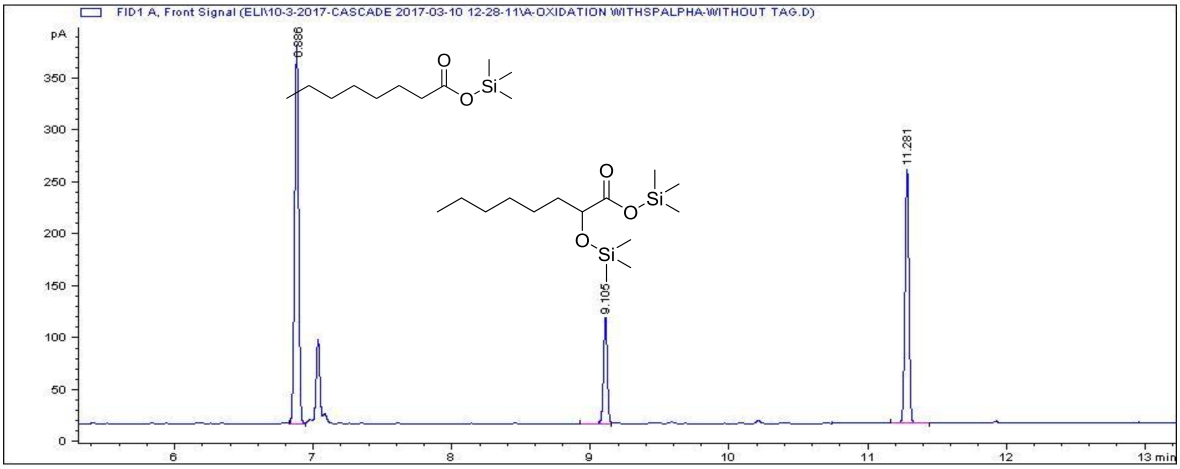

b) 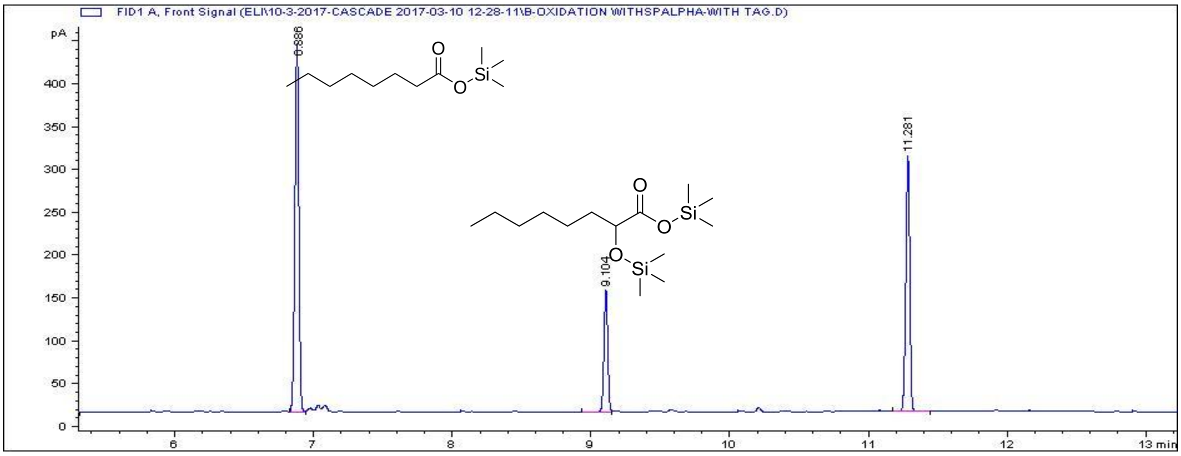

Figure S6. Achiral GC traces from oxidation of 10 mM **1** to **2** using P450$_{SP\alpha}$ a) without tag (cleaved tag in solution) and b) with tag attached.

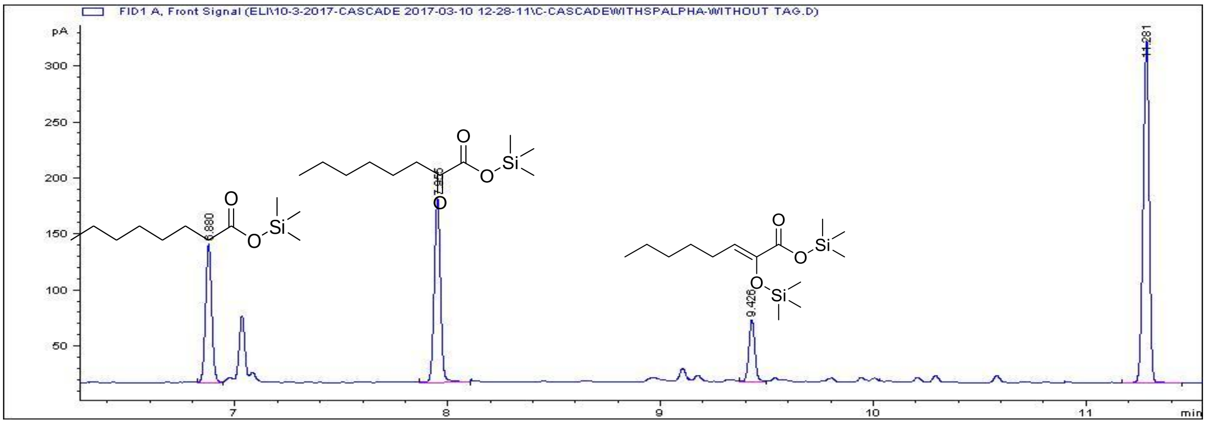

a)

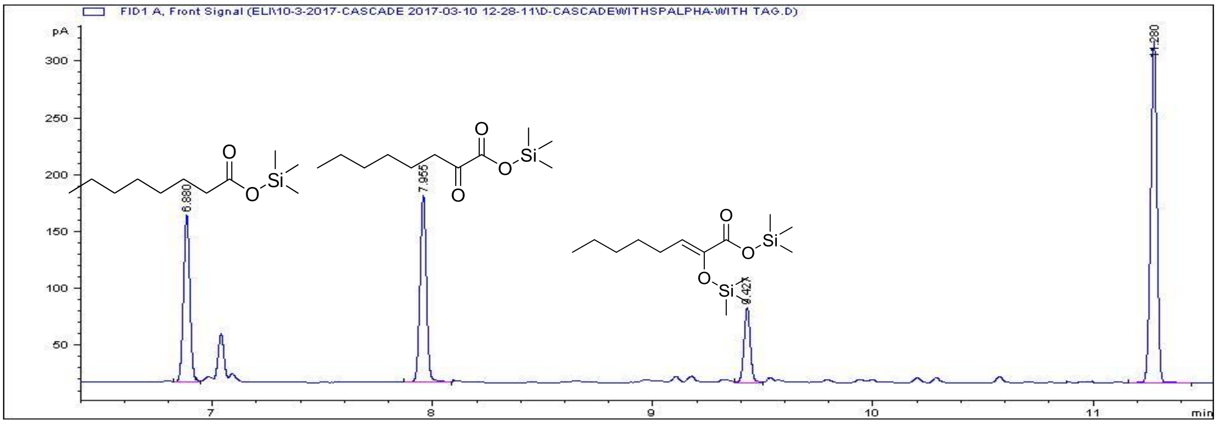

b)

Figure S7. a) Achiral GC traces showing formation of **3** from **1** in the cascade set-up B using $P450_{SP\alpha}$ a) without tag (cleaved tag in solution) and b) with tag attached.

Only small differences between both systems could be observed due to the removal of the tag, confirming the functional activity of the protein in both preparations and indicating that the presence of the free tag in solution was not responsible for side activities.

2.3. Testing cascades A and B

2.3.1. Conversion of octanoic acid (1) to 2-oxooctanoic acid (3) with $P450_{CLA}$/(*S*)-α-HAO

A test cascade was performed on octanoic acid (**1**) using two enzymes in a one-pot cascade consisting of $P450_{CLA}$ and (*S*)-α-HAO combined with a single addition of H_2O_2 at various concentrations. Based on initial data on the enantioselectivity of both reactions — i.e. a moderate enantiopreference for the (*S*)-hydroxylation yielding 36% ee for the (*S*)-hydroxy product and high (*S*)-enantioselectivity in the oxidation of the hydroxy intermediate — the theoretical amount of H_2O_2 required for maximum product formation (full conversion of **1**) equals the amount of (*R*)-hydroxy product formed in the first step, i.e. 32% of the starting material [note: 36% ee corresponds to an enantiomer ratio (*S*)/(*R*) of 68:32]. This translates into a ratio of products (*R*)-**2**/**3** of 32:68 at full H_2O_2 consumption and complete substrate conversion. Thus, the conversion of 10 mM **1** was initiated by addition of 3.2 mM H_2O_2. The oxidant concentration and the amount of (*S*)-α-HAO were additionally varied and correlated with the formation of final products (Table S3). Successful formation of final oxo-product **3** after 12 h could be confirmed in all samples where H_2O_2 and both enzymes were present (entries 1-5, Table S3).

Despite some variations in the recovery (attributed to analytical error), the amount of product **3** obtained from 3.2 mM H_2O_2 corresponds to the expected theoretical value (measured 7.3 mM, with 6.8 mM corresponding to complete H_2O_2-recycling) with maximum allowed formation of (*R*)-**2** (3.3 mM).

Table S3. Conversion of octanoic acid (**1**) to 2-oxooctanoic acid (**3**) through $P450_{CLA}$/(*S*)-α-HAO at various H_2O_2 and (*S*)-α-HAO concentrations (12 h reaction time)

Entry	[H_2O_2] (mM)	(*S*)-α-HAO (μM)	Reaction Time (h)	[1] (mM)	[2] (mM)	[3] (mM)	Ratio 2/3	Recovery %
1	3.2	0.39	12	1.65	3.32	7.33	31:69	123[b]
2	2	0.39	12	3.44	2.19	5.06	30:70	107[b]
3	1	0.39	12	4.95	1.21	2.85	30:70	90
4	0.5	0.39	12	7.54	0.66	1.20	35:65	94
5	0.1	0.39	12	8.39	<0.30[c]	0.61	36:64	93
6	0	0.39	12	8.60	<0.30[c]	n.d.	n.a.	89
7	3.2[a]	0.39	12	9.60	<0.30[c]	n.d.	n.a.	99
8	3.2	0	12	6.03	3.23	n.d.	n.a.	93
9	3.2	0.39	4	6.50	2.58	2.62	50:50	117[b]
10	3.2	0.55	4	6.08	2.68	3.45	44:56	122[b]
11	3.2	0.77	4	5.87	2.46	4.28	36:64	126[b]
12	3.2	0	4	8.45	2.81	n.d.	n.a.	113[b]

[a] No $P450_{CLA}$
[b] Apparent recovery >100% most likely due to the presence of higher amount of enol form of the product (see derivatization product in experimental section) and its expected different response factor on GC-GC/MS compared to the oxo-form (no enol standard available for calibration).
[c] Limit of detection
n.d. not detected; n.a. not applicable.

In the absence of (*S*)-α-HAO, $P450_{CLA}$ could efficiently and regioselectively hydroxylate octanoic acid and the amount of **2** formed equaled the maximum theoretical concentration that can be reached under these conditions (i.e. same as concentration of oxidant used, entry 8, Table S3). In the cascade set-up and independent on the amount of oxidant used, the amount of recovered intermediate **2** was found to correlate with the amount of oxidant used (entries 1-4, Table S3). This, together with the rather steady ratio **2**/**3** (Table S3), confirms that both steps have reached maximum product levels after 12 h (ratio **2**/**3** close to theoretical 32:68). This was confirmed by calculating ee values for **2** after 15 h reaction time: starting from 10 mM **1** and 3.2 mM oxidant, (*R*)-**2** was obtained in 88% ee (83% starting from 0.5 mM oxidant), indicating only minor amount of residual (*S*)-**2** in the cascade reaction mixture (only low amount of (*S*)-α-HAO used), thus corroborating that the oxidation step of (*S*)-**2** was close to completion.

2.3.2. Testing cascade A by using $P450_{CLA}$/GO-LOX/(*S*)-α-HAO

2.3.2.1. Testing different concentrations of $P450_{CLA}$

Cascade A was performed with different $P450_{CLA}$ concentrations under the following conditions: 1 mL buffer (KPi, 100 mM, pH 7.4) containing 10 mM octanoic acid, 10 % EtOH (co-solvent), 0.1 mM FMN, 0.5 mg/mL (S)-α-HAO, 2.0 mg/mL GO-LOX, $P450_{CLA}$ (5- 10 μM) and 1.0 mM H_2O_2. Reactions were performed in duplicates in closed glass-vials at RT and 170 rpm for 24 h and 48 h (Table S4).

Table S4. Conversion of **1** to **3** by using various $P450_{CLA}$ concentrations in the presence of 1.0 mM H_2O_2

**$P450_{CLA}$ concentration (μM)/ reaction time (h)**	**[1] (mM)**	**[2] (mM)**	**[3] (mM)**	**Conversion %**	**Recovery %**
5 μM/24 h	2.3	-	8.0	79.9	103.3
5 μM/48 h	1.9	-	7.6	75.8	94.5
6 μM/24 h	2.1	-	8.6	85.8	106.8
7 μM/24 h	2.1	-	8.8	88.4	109.7
7 μM/48 h	1.4	-	7.7	77.1	90.8
8 μM/24 h	1.9	-	8.7	87.1	105.9
9 μM/24 h	1.6	-	8.5	85.3	101.0
10 μM/24 h	1.4	-	8.9	88.6	102.2
Control-1 (without $P450_{CLA}$)	8.9	-	0	0	88.5
Control-2 (without GO-LOX)	6.6	1.1	2.0	31.3	93.0
Control-3 (without (*S*)-α-HAO)	5.9	0.9	2.3	33.5	97.0

2.3.2.2. Testing different concentrations of GO-LOX

The effect of GO-LOX concentration was tested in cascade A using following reaction conditions: 1 mL buffer (KPi, 100 mM, pH 7.4) containing 10 mM octanoic acid, 10 % EtOH, 0.1 mM FMN, 0.5 mg/mL (S)-α-HAO, GO-LOX (0.2, 0.5, 1.0 and 2.0 mg/mL), 5 μM $P450_{CLA}$ and 3.0 mM H_2O_2. Reactions were performed in closed glass-vials at RT and 170 rpm for 24 h (Table S5).

Table S5. Conversion of octanoic acid (**1**) to 2-oxooctanoic acid (**3**) by using various GO-LOX concentrations in the presence of 3.0 mM H_2O_2

**GO-LOX concentration (mg/mL)**	**[1] (mM)**	**[3] (mM)**	**Conversion %**	**Recovery %**
0.2	0.3	9.8	97.7	100.2
0.5	0.3	9.2	91.7	94.3
1.0	0.3	9.4	94.1	96.7
2.0	0.4	9.8	97.7	101.3

Results revealed that GO-LOX concentration had only a minimal influence on conversion.

2.3.2.3. Testing cascade A at different temperatures in presence of 0.2 or 0.5 mM H_2O_2

Temperature effects were tested by performing cascade A under the following conditions: 1 mL buffer (KPi, 100 mM, pH 7.4) containing 10 mM octanoic acid, 10 % EtOH (co-solvent), 0.1 mM FMN, 0.5 mg/mL (S)-α-HAO, 0.2 mg/mL GO-LOX, 5 µM $P450_{CLA}$ and H_2O_2 at varying concentrations (0.2 and 0.5 mM). Cascade reactions were performed in closed glass-vials at 4 ºC and 170 rpm shaking for 24 h and 48 h (Table S6).

Table S6. Conversion of octanoic acid (**1**) to 2-oxooctanoic acid (**3**) by using 0.2 mg/mL GO-LOX in the presence of 0.2 or 0.5 mM H_2O_2 at 4 ºC, compared to data obtained at room temperature (TON)

**[H_2O_2]/reaction time**	**[1] (mM)**	**[3] (mM)**	**Conversion %**	**Recovery %**	**$TON_{H_2O_2}$ (4 ºC)**	**$TON_{H_2O_2}$ (RT)**
0.2 mM /24 h	7.3	1.7	16.6	89.2	8.3	14.9
0.5 mM /24 h	5.6	2.8	28.1	84.1	5.6	11.4
0.2 mM /48 h	5.7	1.6	16.5	73.3	8.3	15.1
0.5 mM /48 h	4.4	3.8	38.3	81.9	7.7	11.7

Results reveal that higher conversions were achieved at room temperature compared to 4 ºC and, as expected by using higher H_2O_2 concentration, higher conversions were achieved at 0.5 mM.

In addition to room temperature and 4 ºC, the cascade was performed at 30 ºC as well. Due to enzyme inactivation at this temperature, no conversion was observed in all tested conditions.

2.3.3. Testing cascade B by using $P450_{SP\alpha}$/(*S*)-α-HAO

Cascade B was tested with different concentrations of $P450_{SP\alpha}$ using following reaction conditions: 1 mL buffer (KPi, 100 mM, pH 7.4) containing 10 mM octanoic acid, 10 % EtOH (co-solvent), 0.1 mM FMN, 1.0 mg/mL (S)-α-HAO, $P450_{SP\alpha}$ (3-10 µM) and 1.0 mM H_2O_2. Cascade reactions were performed in closed glass-vials at RT and 170 rpm shaking for 24 h (Table S7).

Table S7. Conversion of octanoic acid (**1**) to 2-oxooctanoic acid (**3**) by using various $P450_{SP\alpha}$ concentration in the presence of 1.0 mM H_2O_2

**$P450_{SP\alpha}$ concentration (µM)**	**[1] (mM)**	**[3] (mM)**	**Conversion %**	**Recovery %**
3	5.5	5.2	51.6	106.4
5	3.6	5.9	59.2	95.6
8	4.5	5.3	53.2	98.4
10	4.5	5.1	51.5	96.4

Best conversion was achieved by using 5 µM of $P450_{SP\alpha}$, and this concentration was used for further experiments.

2.4. Scale-up experiments

2.4.1. Cascade A

Upscaling cascade A was performed in 60 mL buffer (KPi, 100 mM, pH 7.4) containing 10 mM octanoic acid (**1**), 10% EtOH (co-solvent), 5 µM $P450_{CLA}$, 0.1 mM FMN, 24 µM (*S*)-α-HAO, 15 µM GO-LOX and 1.0 mM H_2O_2. After 5 h reaction time, 1.0 mM H_2O_2 was added to the reaction mixture and stirring was continued for 17 h at room temperature. Work-up consisted in acidification with 5 M HCl (5 mL) followed by extraction with ethyl acetate (3 x 45 mL) with centrifugation (3 × 10 min at 4,688 x g) for phase separation. The combined organic phases were dried over Na_2SO_4 and the solvent was evaporated under reduced pressure, yielding 70 mg (74% of isolated yield) of the final 2-oxooctanoic acid (**3**) with a purity of 90%, as confirmed by ^{1}H-NMR (see Figure S13). No further purification was performed and achiral GC analysis revealed a conversion of 98% (according to procedure described in 3.1).

^{1}H-NMR (300 MHz, $CDCl_3$): δ 7.07 (brs, 1H), 2.92 (t, *J* = 7.3 Hz, 2H), 1.67 – 1.60 (m, 2H), 1.38 – 1.25 (m, 6H), 0.90 – 0.86 (m, 3H). ; in accordance with spectrum of commercially available sample (Figure 16).

2.4.2. Cascade B

Upscaling cascade B was performed in 35 mL buffer (KPi, 100 mM, pH 7.4), containing 5 µM $P450_{sp\alpha}$, 10 mM octanoic acid (**1**), 10% EtOH (co-solvent), 0.1 mM FMN, 24 µM (*S*)-α-HAO and 1.0 mM H_2O_2. After 5 h from starting the reaction, 1.0 mM H_2O_2 was added to the reaction mixture and stirring was continued for 17 h. Work-up consisted in acidification with 5 M HCl (3 mL) followed by extraction with ethyl acetate (3 x 20 mL) with centrifugation (3×10 min at 4,688 x g) for phase separation. The combined organic phases were dried over Na_2SO_4 and the solvent was evaporated under reduced pressure, yielding 50 mg (91% isolated yield) of 2-oxooctanoic acid (**3**) with a purity of 93%, as confirmed by ^{1}H-NMR and ^{13}C-NMR (see Figures S14 & S15). No further purification was performed and achiral GC analysis revealed a conversion of 97% (according to procedure described in 3.1).

^{1}H-NMR (300 MHz, $CDCl_3$): δ 7.83 (brs, 1H), 2.92 (t, *J* = 7.3 Hz, 2H), 1.67 – 1.62 (m, 2H), 1.35 – 1.26 (m, 6H), 0.90 – 0.85 (m, 3H); ^{13}C-NMR (75 MHz, $CDCl_3$) δ 195.8, 160.3, 37.6, 31.4, 28.6, 23.0, 22.4, 14.0; in accordance with spectra of commercially available sample (Figures 16-17).

2.5. Conversion of C6:0, C7:0 and C10:0 fatty acids in cascade set-ups A and B

Additional substrates including hexanoic acid (C6), heptanoic acid (C7) and decanoic acid (C10) were tested in both cascades. The reaction was performed in 1 mL buffer (KPi, 100 mM, pH 7.4), containing 10 mM substrate (C6, C7 and C10), 10% EtOH (co-solvent), 5 µM P450 ($P450_{CLA}$ in cascade A and $P450_{SP\alpha}$ in cascade B), 0.1 mM FMN, 24 µM (*S*)-α-HAO, 15 µM GO-LOX in case of cascade A, and H_2O_2 at varying concentrations (Table S8). Cascade reactions were performed in duplicates in closed glass-vials at room temperature and 170 rpm for 24 h. Work-up consisted in acidification with 5 M HCl (100 µL) followed by extraction with ethyl acetate (2 x 0.5 mL) spiked with dodecanoic acid (5 mM) as internal standard. Derivatization for GC and GC-MS analysis was performed by silylation of the organic phase using BSTFA (Figure S16-S35). Calibration curves were generated using standards of the substrates.

Table S8. Conversion of 10 mM substrate (hexanoic, heptanoic and decanoic acid). N.d.: not determined.

**Entry**		**[H_2O_2] (mM)**	**Remaining substrate (mM)**		**Calculated conversion%**	
			**Cascade A**	**Cascade B**	**Cascade A**	**Cascade B**
1	**C6:0**	1+1	n.d.	n.d.	n.d.	n.d.
2		3	5.0	6.8	50	32
3	**C7:0**	1+1	3.1	6.6	69	34
4		3	2.7	7.1	73	29
5	**C10:0**	1+1	3.3	2.0	67	80
6		3	2.7	0.7	73	93

For all tested substrates (C6:0, C7:0 and C10:0), 2-ketoacid products were detected as sole products, and thus conversions were calculated based on substrate consumption. GC and GC-MS chromatograms (Figure S16-S35) show formation of the final 2-ketoacids products in each case, detected as the corresponding silylated derivatives. The results reveal that both cascade set-ups accept other substrates with moderate to high conversion level (cascades not optimized). As the chain length of the substrate increases, higher conversions are achieved by using the cascade set-up B (two-enzyme). For shorter substrates (C6:0 and C7:0), the three-enzyme set-up appears more suited. In summary, the internal H_2O_2 recycling protocol is applicable to a variety of fatty acids.

3. Analytical methods

3.1. GC, GC-MS and NMR

Silylation was found to be the most suitable derivatization method (quantitative yield to corresponding silylated substrate, hydroxy- and oxo-products). Silylation of final oxo-product delivered a mixture of derivatized oxo-acid and derivatized enol-form of the oxo-acid (Figure S8).

After derivatization as described under 1.4.3, GC-MS measurements were carried out on a 7890A GC System (Agilent Technologies, Santa Clara, CA, USA), equipped with a 5975C mass selective detector and an HP-5MS column (5% phenylmethylsiloxane, 30 m x 320 µm, 0.25 µm film, J&W Scientific, Agilent Technologies) using He as carrier gas. Injector temperature: 250 °C; injection volume: 1 µL; flow rate: 0.7 mL/min; temperature program 1: 100 °C, hold 0.5 min, 10 °C/min to 300 °C; EI mode, energy 70 eV, MS Source: 230 °C, MS Quadrupole: 150 °C.

GC measurements were performed on an Agilent Technologies 7890 A GC system equipped with a FID-detector and a 7693A Injector in combination with a 7693 Series Autosampler and using a HP-5 column (30 m x 320 µm, 0.25 µm film, J&W Scientific, Agilent Technologies) using He as carrier gas. Injector temperature: 250 °C; injection volume: 5 µL; flow rate: 0.7 mL/min; temperature program 1: 100 °C, hold time 0.5 min, 10 °C/min to 300 °C). For quantification of the final product, both peaks corresponding to oxo-product **2** arising from oxo-compound and enol form were integrated (see Figures S8 and S11). It should be noted that the extent of tautomerism yielding the enol form could not be controlled (in standards and reaction samples). Although both peaks were integrated for calculation, some deviation in the concentration of the

final product is therefore possible (no individual standard available for the enol form and likely different response factors for both forms).

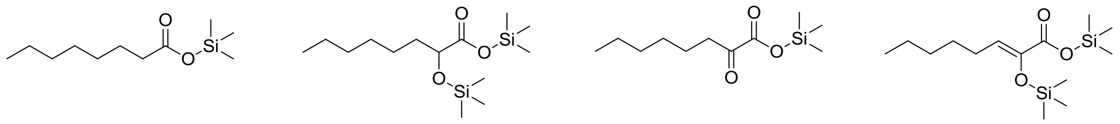

Figure S8. Silylated compounds obtained from derivatization procedure

^{1}H and ^{13}C-NMR spectra were recorded using a Bruker AVANCE III 300 MHz spectrometer using a 5mm BBO probe at 300 K. Chemical shifts (*δ*) are given in parts per million (ppm) relative to TMS (δ = 0 ppm) or to the residual solvent signal, and coupling constants (*J*) are reported in Hertz (Hz).

3.2. Determination of *ee* value of 2-hydroxyoctanoic acid on chiral GC

Derivatization using ethyl chloroformate/methanol was employed using the following procedure: Reaction samples (1 mL) were shock-frozen and lyophilized, the resulting residue was taken up in 700 µL MeOH containing 5% DMAP, and 150 µL ethyl chloroformate was added. The reaction was performed at 50 °C and 700 rpm for 1 h. After solvent removal (speedvac or air flow), 700 µL of 2% aq. HCl was added and extraction was performed with EtOAc (2 x 500 µL). After drying of the combined organic phases over Na_2SO_4, samples were analyzed on GC. GC measurements were performed on an Agilent Technologies 7890 A GC system equipped with a FID-detector and a 7693A Injector in combination with a 7693 Series Autosampler and using a Chirasil ChiralDexCB column (25 m x 320 µm, 0.25 µm film) using H_2 as carrier gas. Injector temperature: 250 °C; injection volume: 1 µL; flow rate: 1.3 mL/min; detector temperature: 250 °C; temperature program: 100 °C, hold 1 min, 10 °C/ min to 130 °C, hold 5 min, 10 °C/min, 180 °C, hold 1 min. Retention times: Derivatized (*R*)-**2**: 7.36 min; derivatized (*S*)-**2**: 7.64 min.

3.3. GC/ GC-MS chromatograms and NMRs

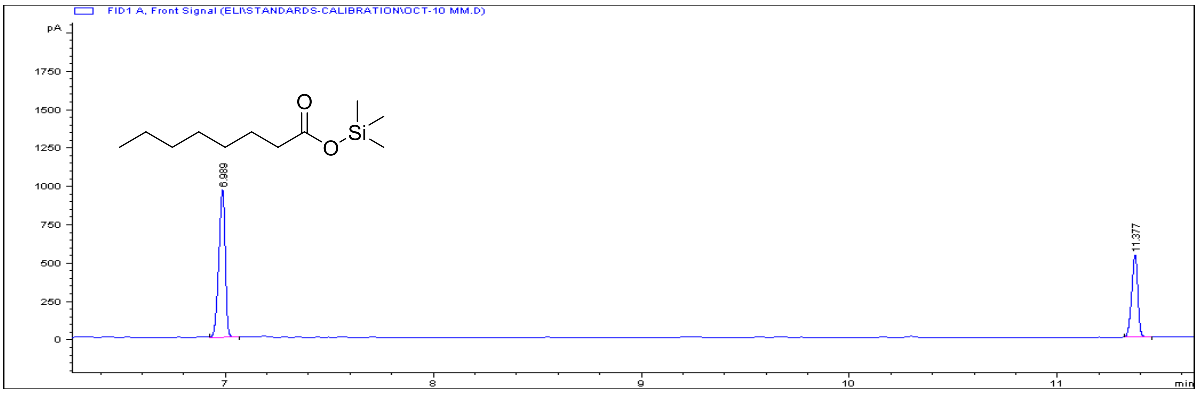

Figure S9. Achiral GC chromatogram of derivatized octanoic acid **1** (internal standard dodecanoic acid with t_{ret} 11.4 min)

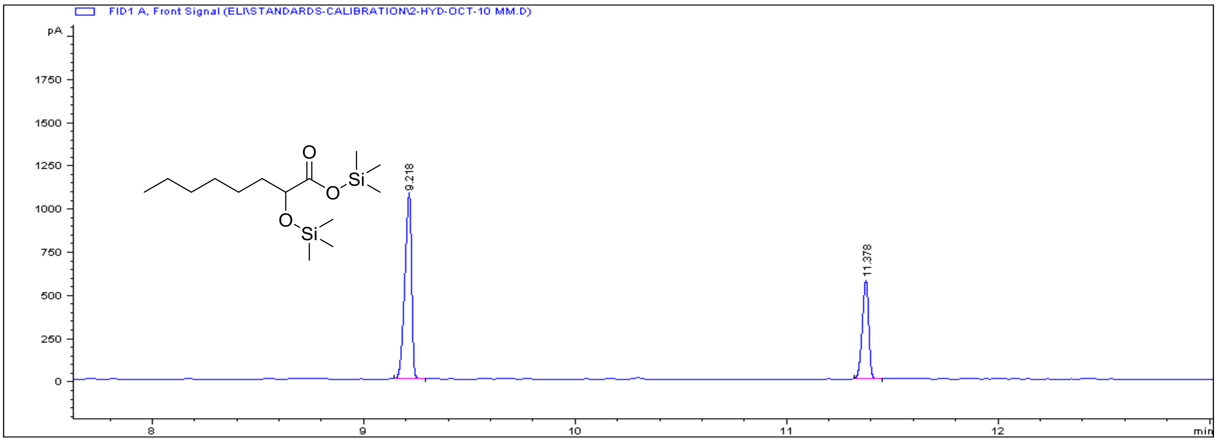

Figure S10. Achiral GC chromatogram of derivatized 2-hydroxyoctanoic acid **2** (internal standard dodecanoic acid with t_{ret} 11.4 min)

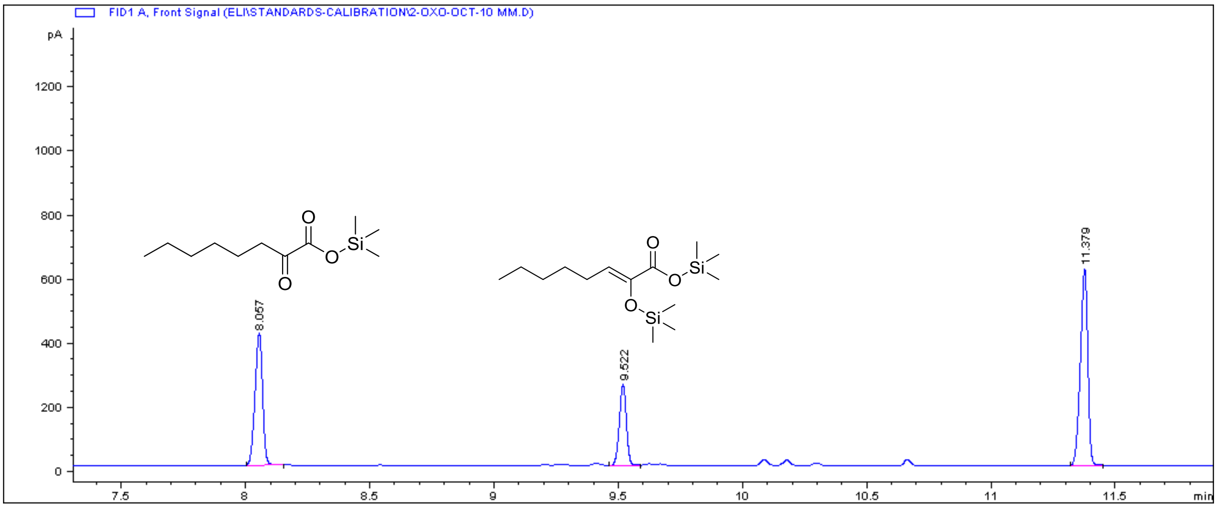

Figure S11. Achiral GC chromatogram of derivatized 2-oxo octanoic acid **3** (internal standard dodecanoic acid with t_{ret} 11.4 min)

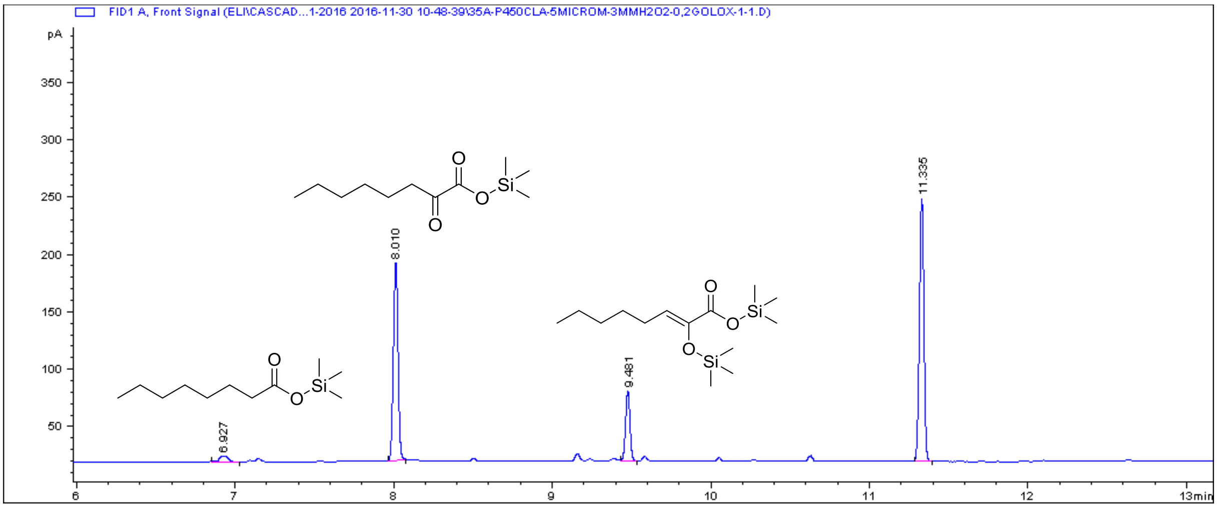

Figure S12. Achiral GC chromatogram obtained from cascade A using 3.0 mM H_2O_2 as described in Table 1 of main text (internal standard dodecanoic acid with t_{ret} 11.4 min)

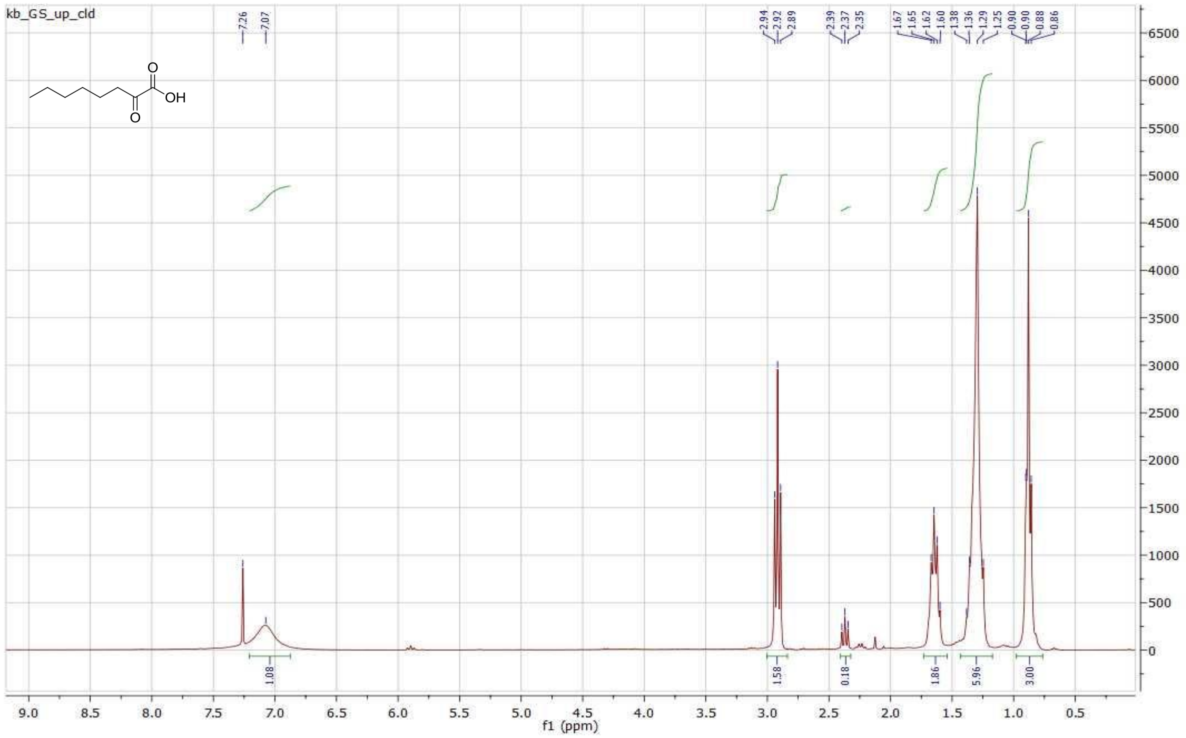

Figure S13. ^{1}H-NMR of 2-oxo octanoic acid (**3**) obtained from the upscaling of cascade A (triplet at δ 2.37 ppm is related to residual substrate)

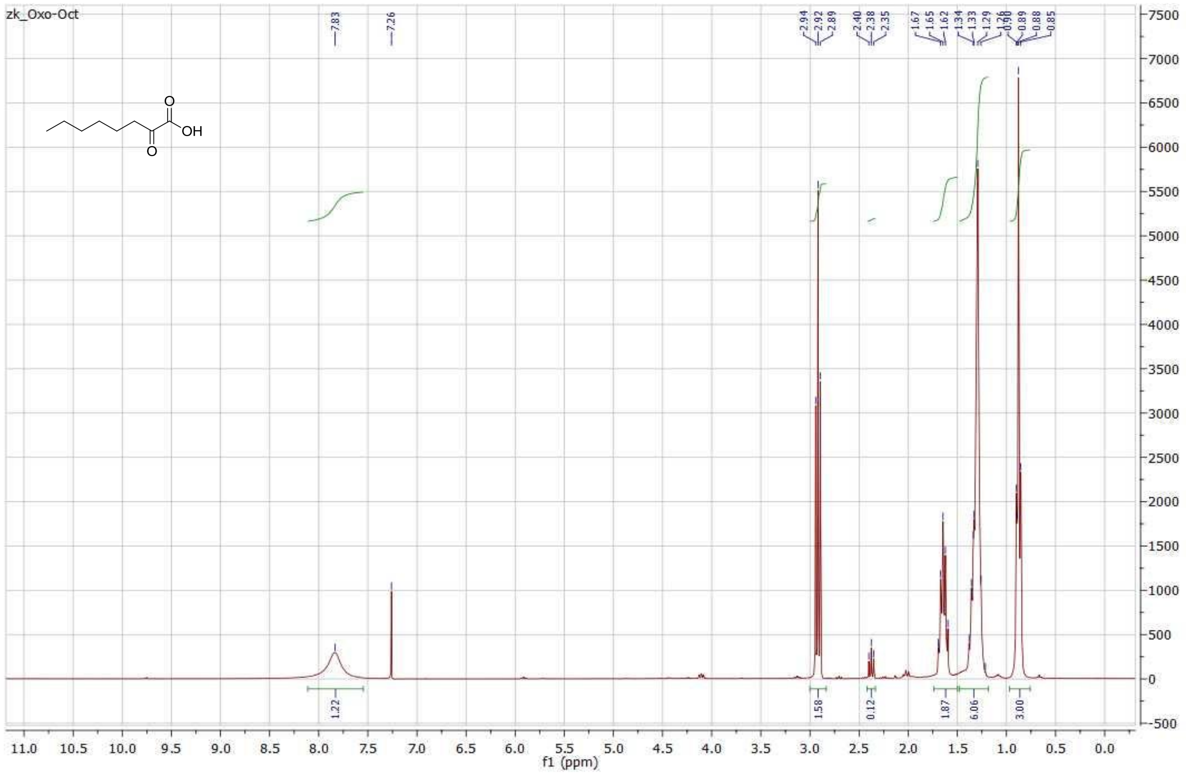

Figure S14. ^{1}H-NMR of 2-oxo octanoic acid (**3**) obtained from the upscaling of cascade B (triplet at δ 2.38 ppm is related to residual substrate)

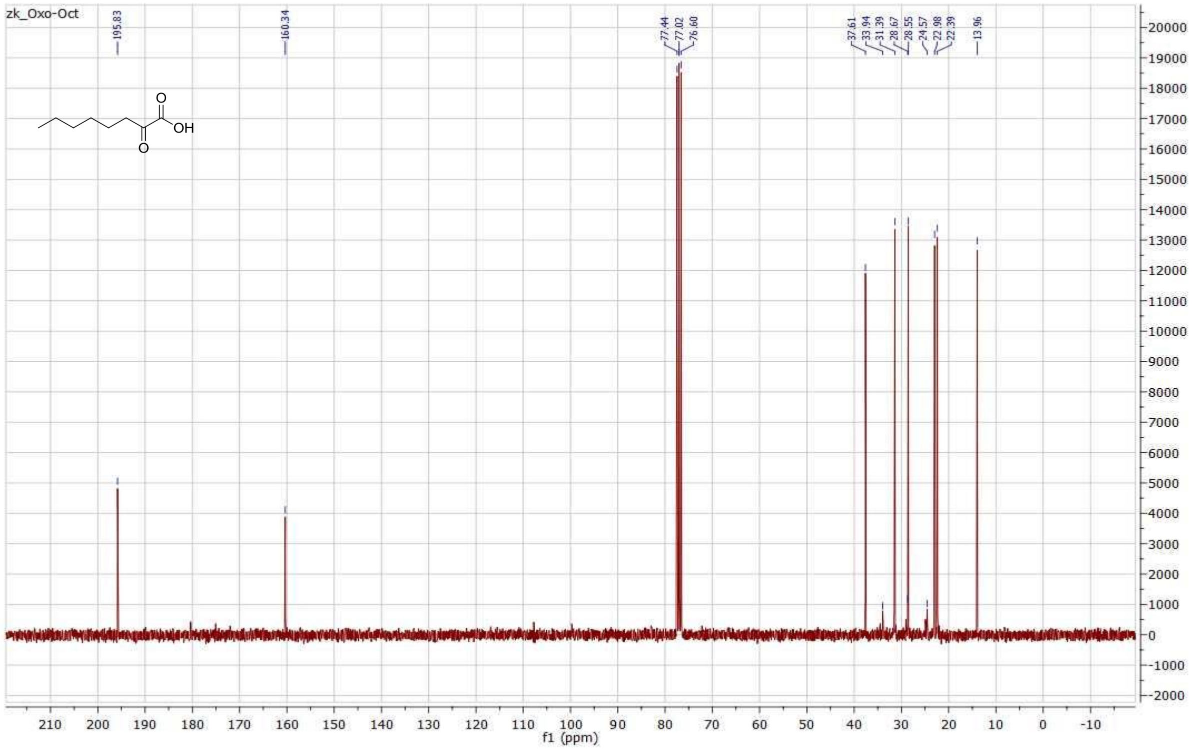

Figure S15. ^{13}C-NMR of 2-oxo octanoic acid (**3**) obtained from the upscaling of cascade B

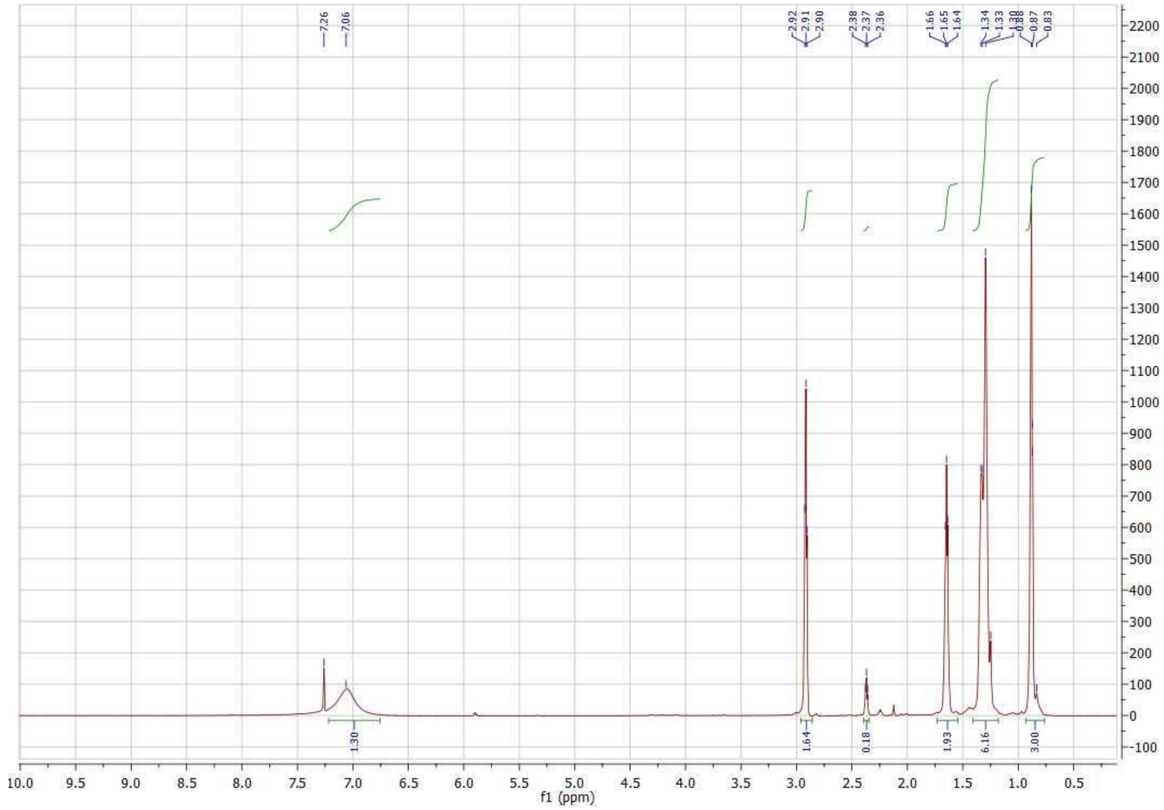

Figure S16. ^{1}H-NMR of 2-oxo octanoic acid purchased from Sigma as ≥99% purity sample.

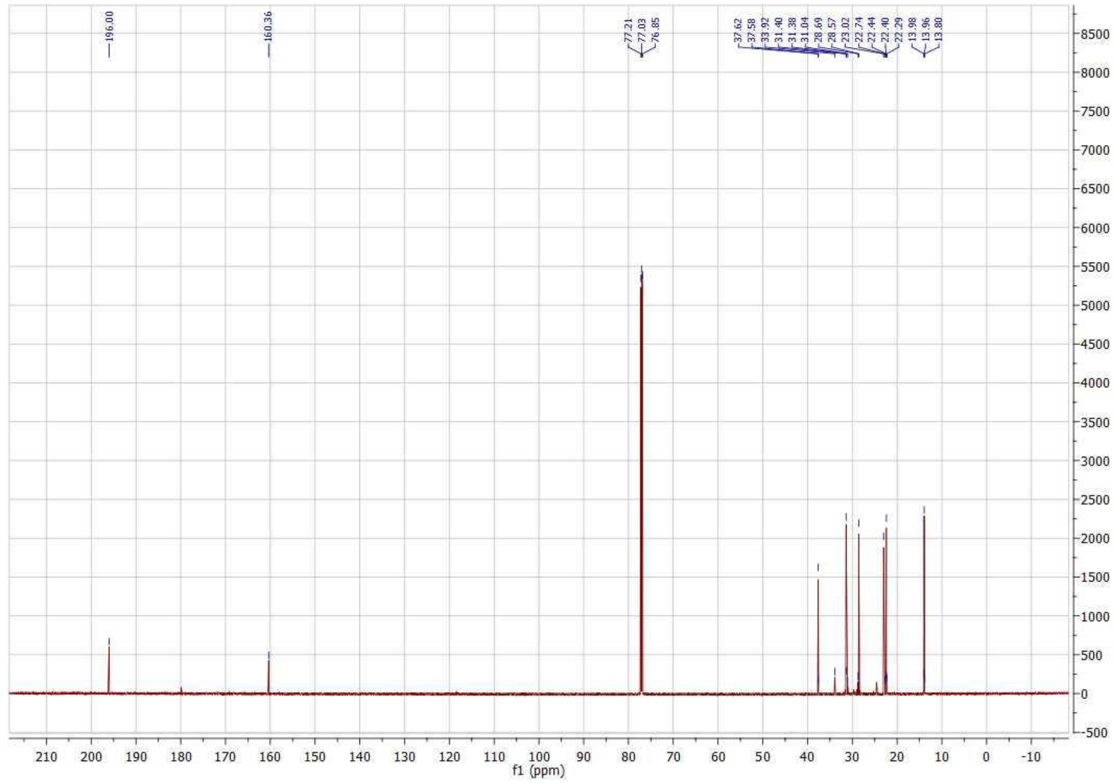

Figure S17. ^{13}C-NMR of 2-oxo octanoic acid purchased from Sigma as ≥99% purity sample.

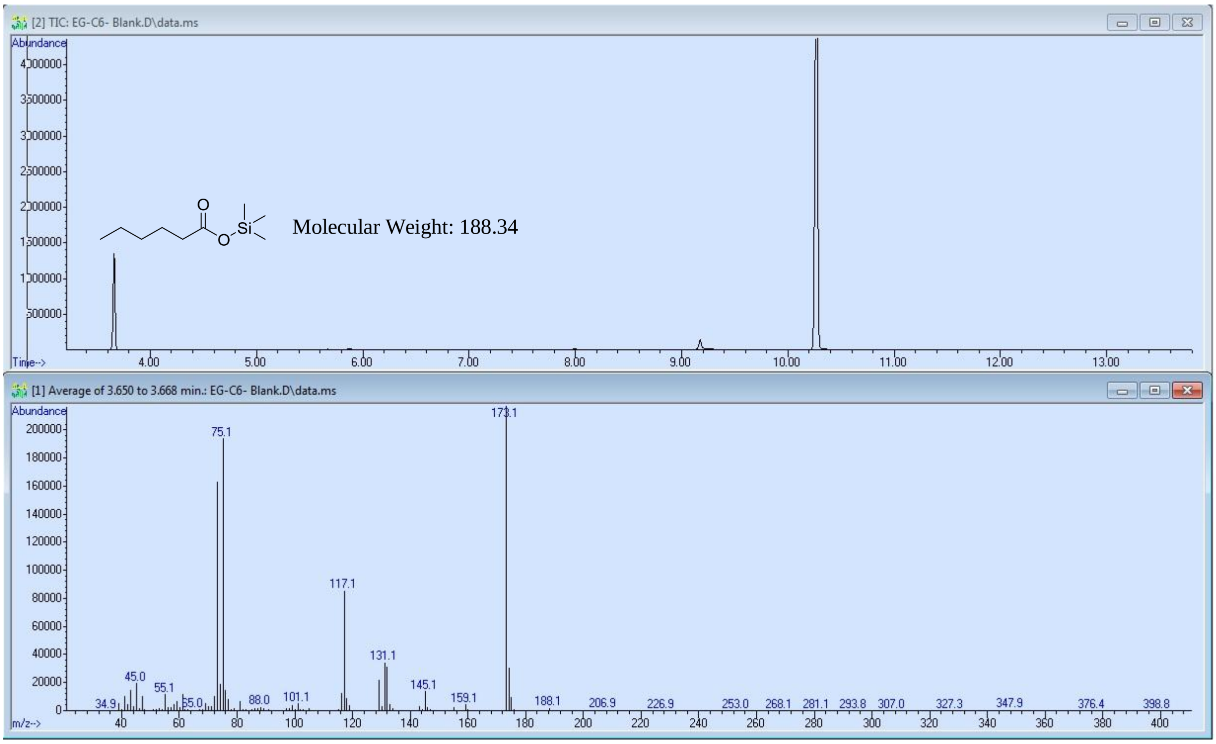

Figure S18. GC-MS chromatogram of derivatized hexanoic acid with dodecanoic acid (t_{ret} 10.3 min) as internal standard

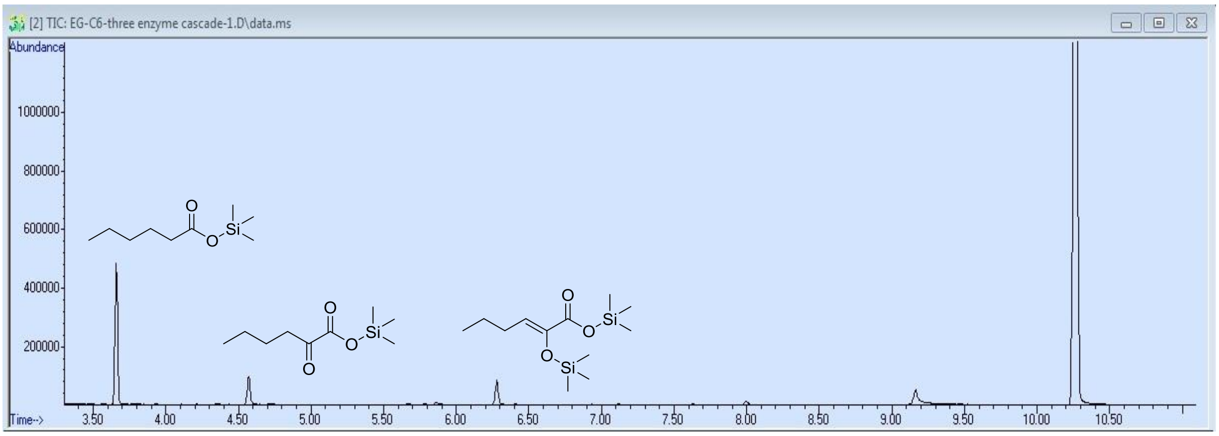

Figure S19. GC-MS chromatogram obtained from cascade A using 3.0 mM H_2O_2 and hexanoic acid as substrate (as described in Table S8, entry 2) with dodecanoic acid (t_{ret} 10.3 min) as internal standard

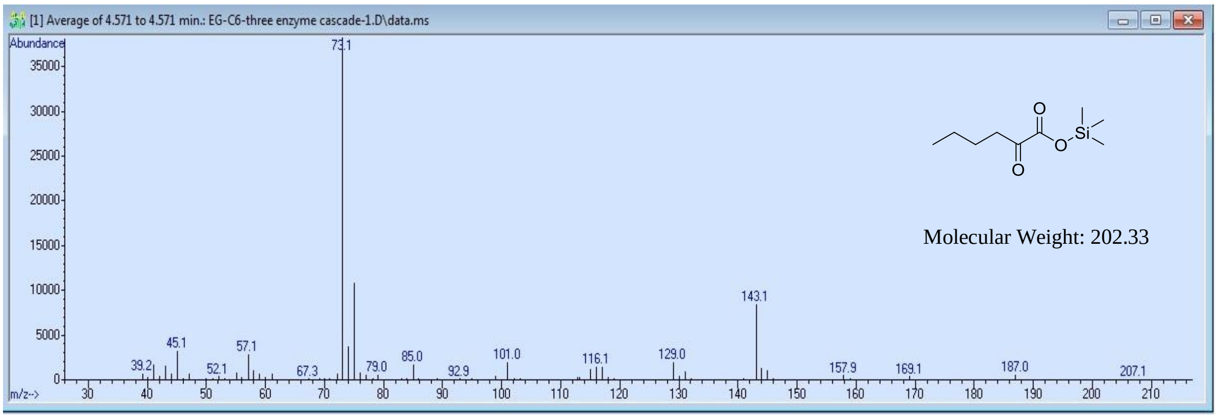

Figure S20. MS of peak of derivatized 2-oxohexanoic acid at 4.5 min from Figure S19

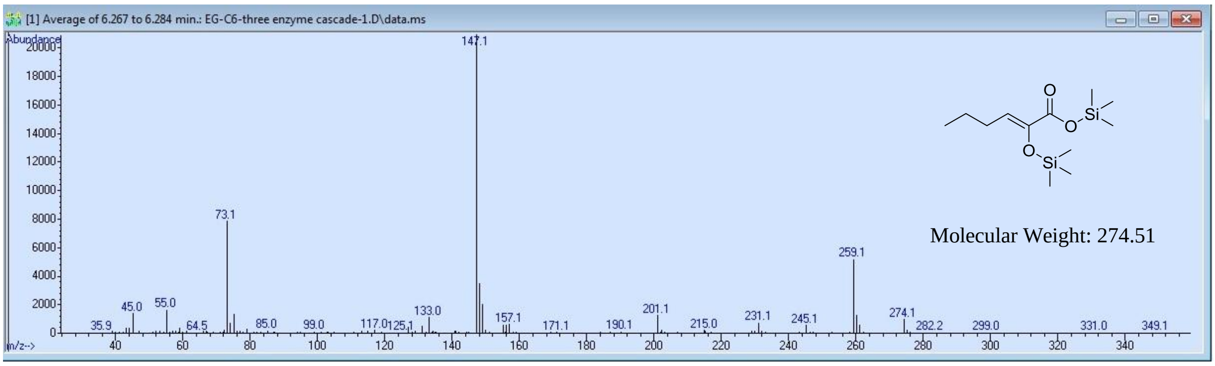

Figure S21. MS of peak of derivatized 2-oxohexanoic acid (enol) at 6.3 min from Figure S19

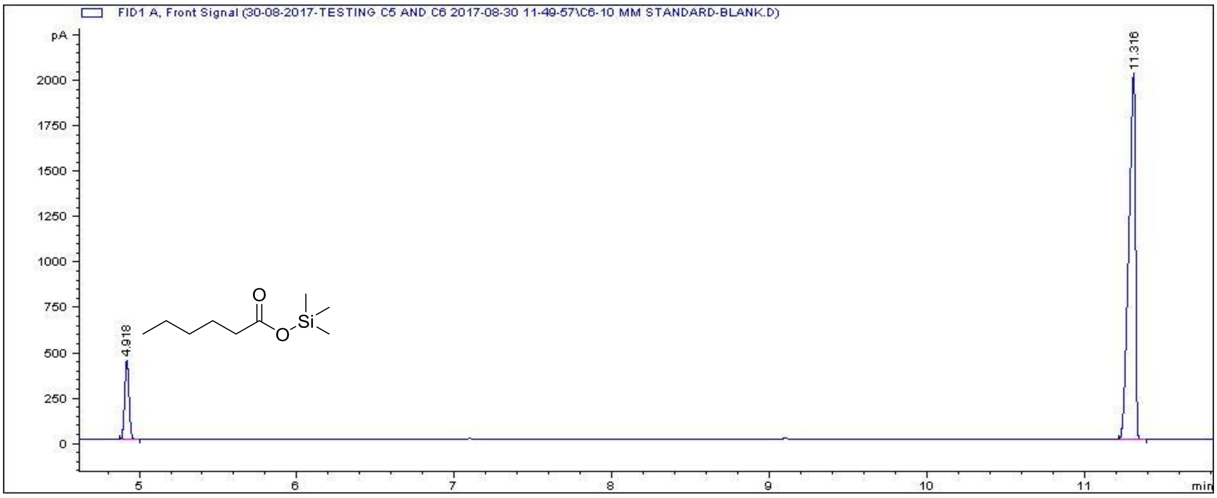

Figure S22. Achiral GC chromatogram of derivatized hexanoic acid with dodecanoic acid (t_{ret} 11.3 min) as internal standard

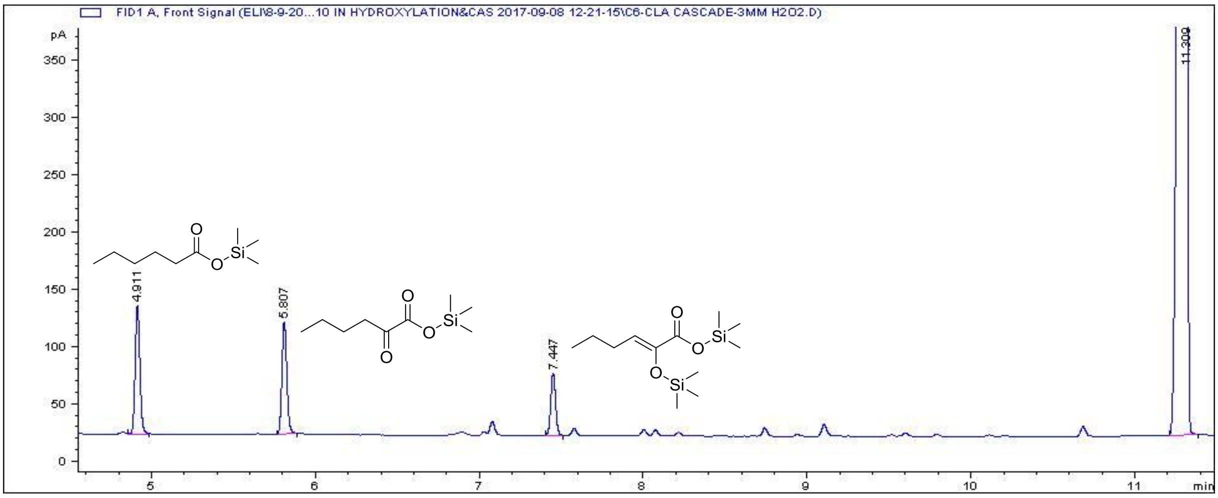

Figure S23. Achiral GC chromatogram obtained from cascade A using 3.0 mM H_2O_2 and hexanoic acid as substrate (as described in Table S8, entry 2) with dodecanoic acid (t_{ret} 11.3 min) as internal standard

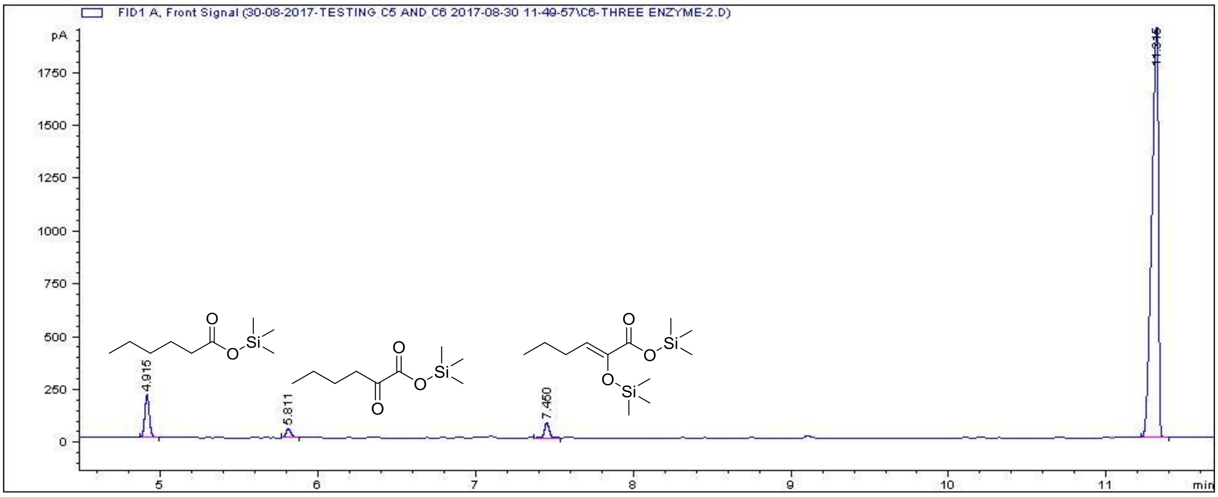

Figure S24. Achiral GC chromatogram obtained from cascade B using 3.0 mM H_2O_2 and hexanoic acid as substrate (as described in Table S8, entry 2) with dodecanoic acid (t_{ret} 11.3 min) as internal standard

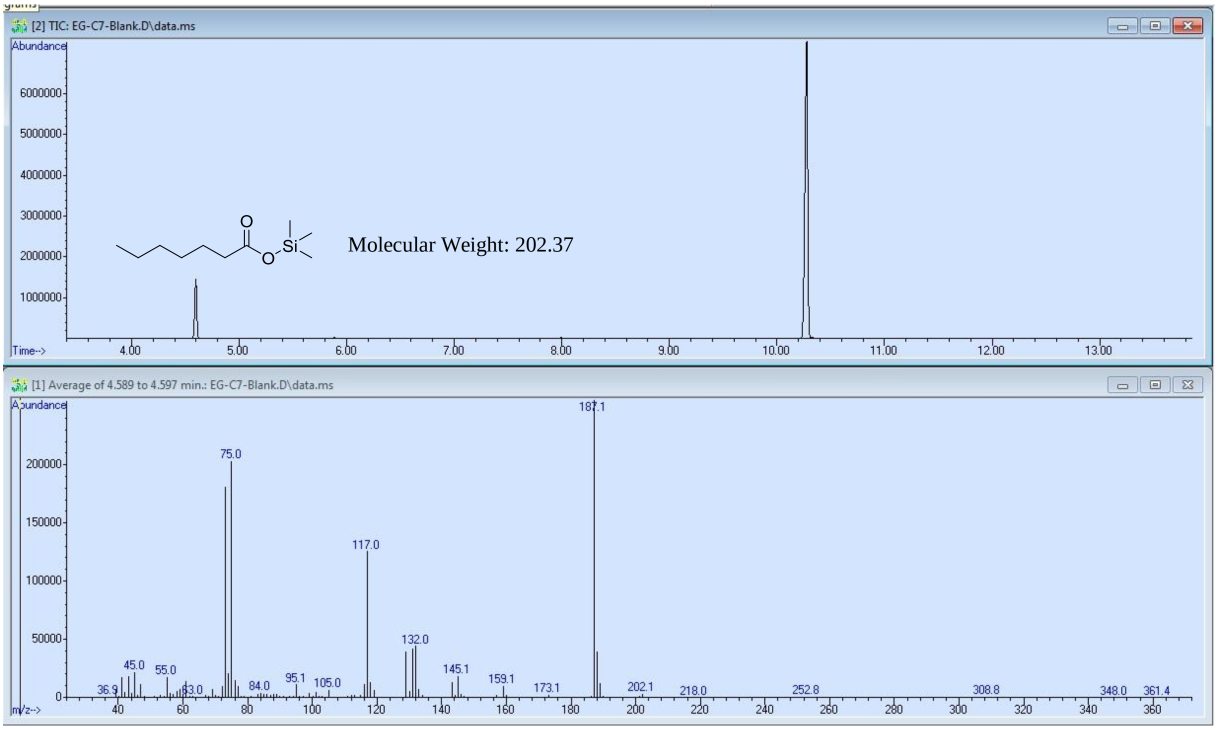

Figure S25. GC-MS chromatogram of derivatized heptanoic acid with dodecanoic acid (t_{ret} 10.3 min) as internal standard

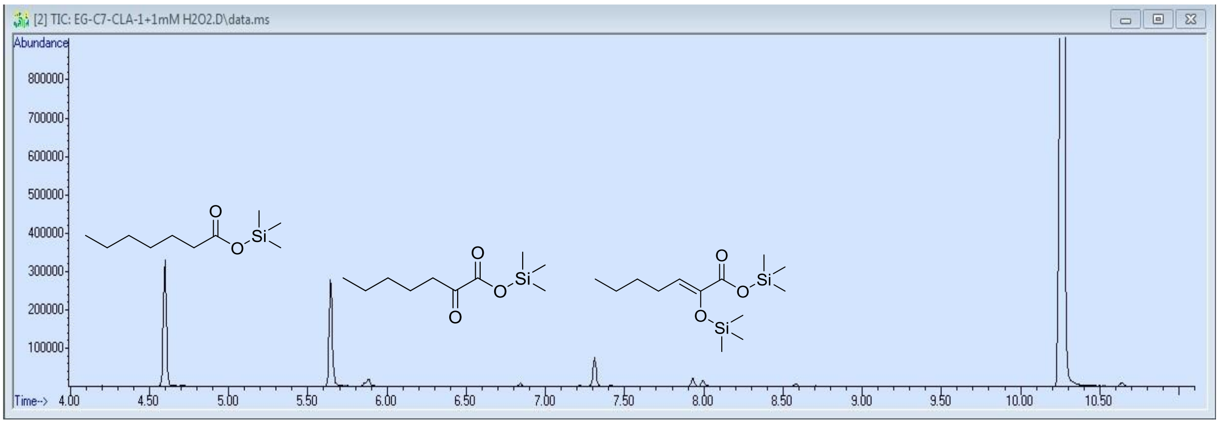

Figure S26. GC-MS chromatogram obtained from cascade A using 1.0+1.0 mM H_2O_2 and heptanoic acid as substrate (as described in Table S8, entry 3) with dodecanoic acid (t_{ret} 10.3 min) as internal standard

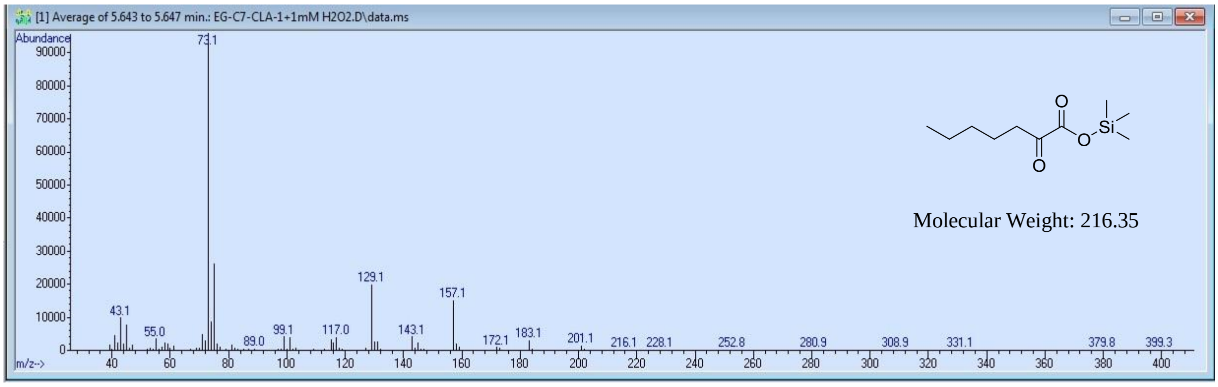

Figure S27. MS of peak of derivatized 2-oxoheptanoic acid at 5.6 min from Figure S26

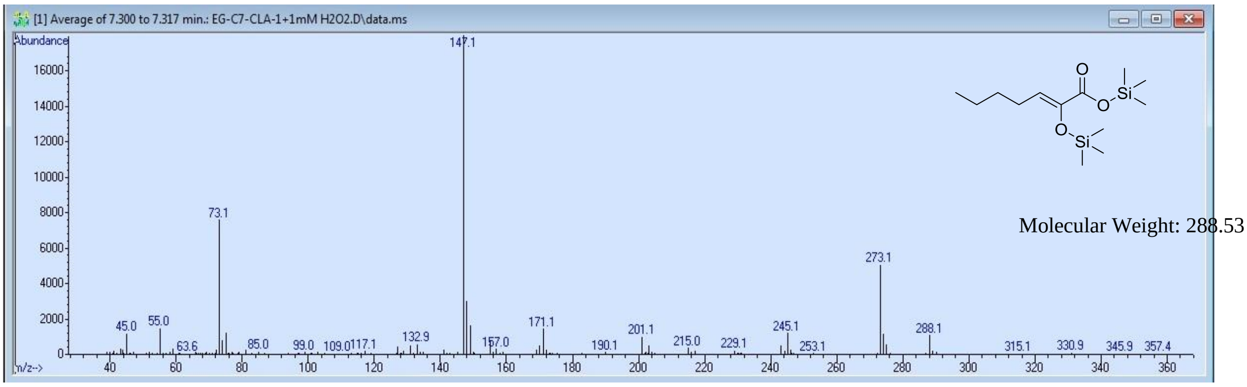

Figure S28. MS of peak of derivatized 2-oxoheptanoic acid (enol) at 7.3 min from Figure S26

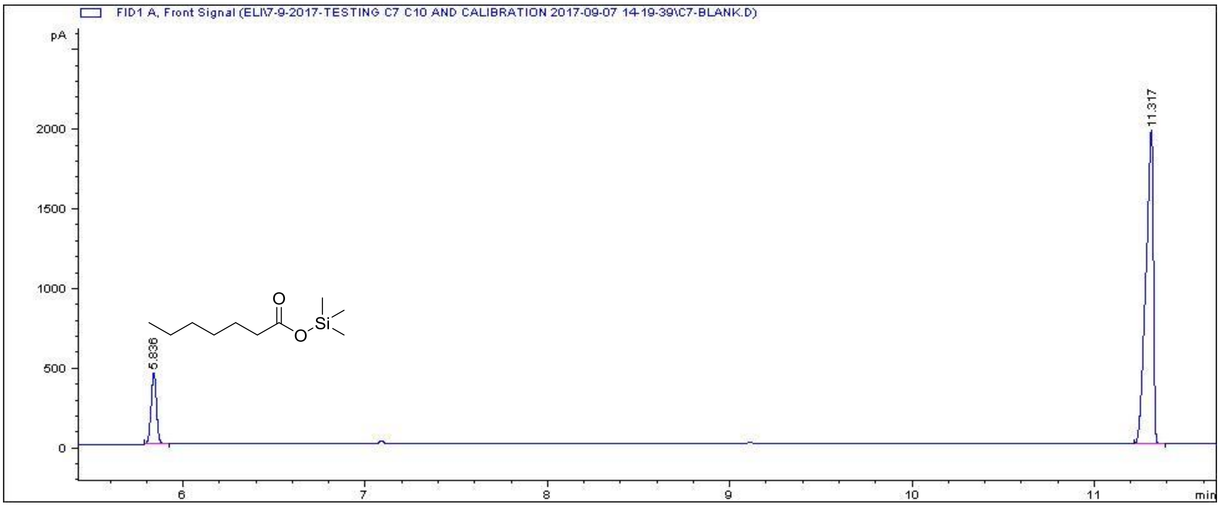

Figure S29. Achiral GC chromatogram of derivatized heptanoic acid with dodecanoic acid (t_{ret} 11.3 min) as internal standard

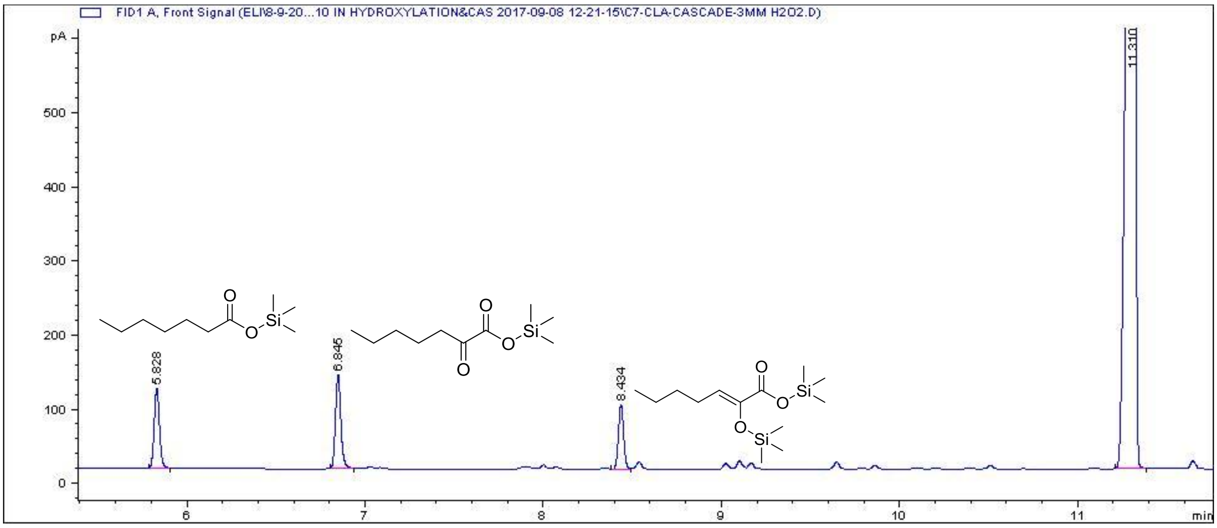

Figure S30. Achiral GC chromatogram obtained from cascade A using 3.0 mM H_2O_2 and heptanoic acid as substrate (as described in Table S8, entry 4)

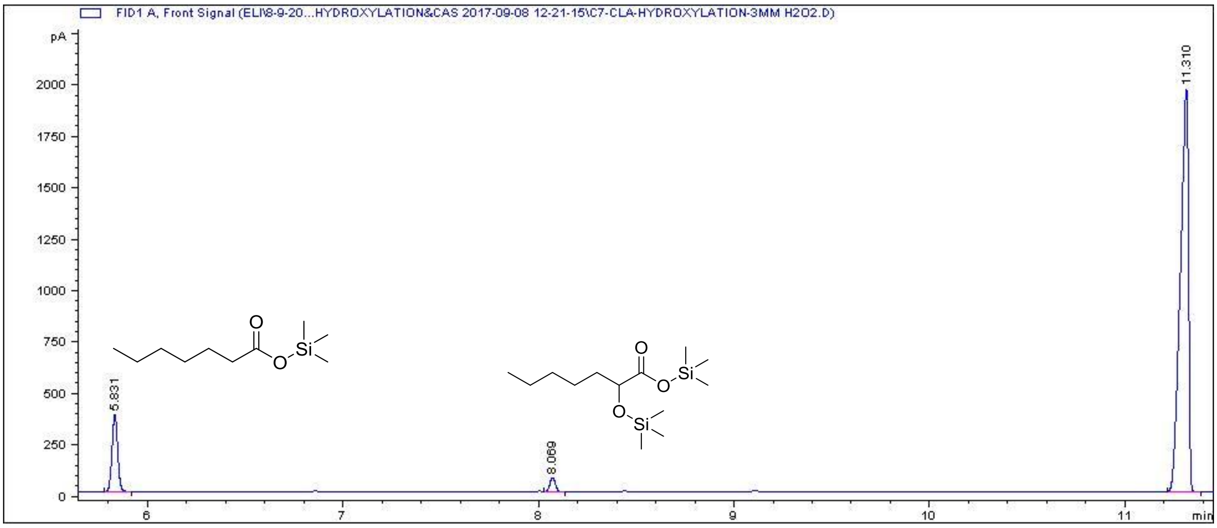

Figure S31. Achiral GC chromatogram obtained from hydroxylation step of the cascade A using 3.0 mM H_2O_2 and heptanoic acid as substrate

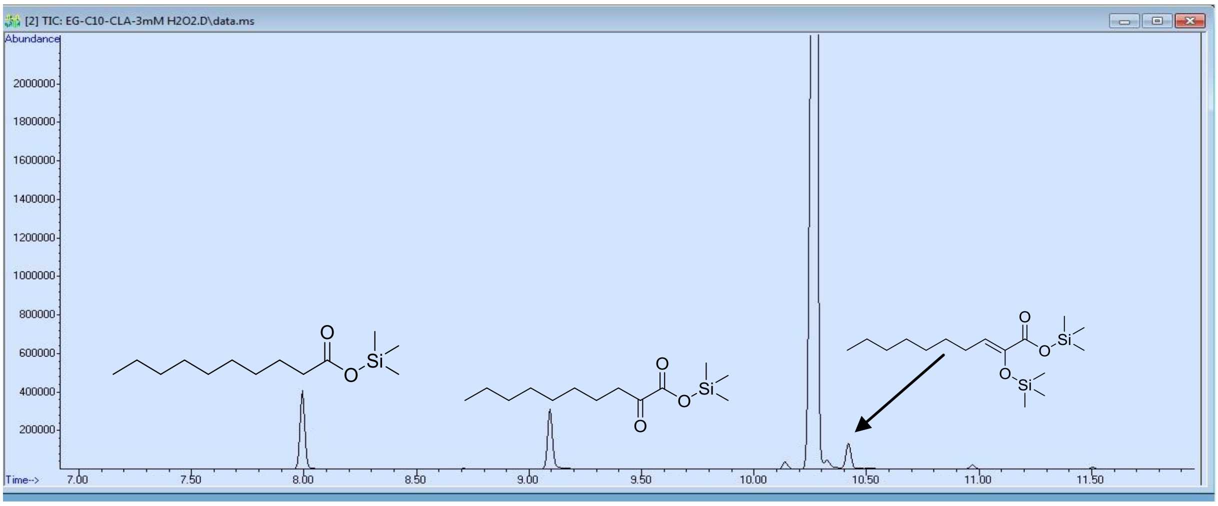

Figure S32. GC-MS chromatogram obtained from cascade A using 3.0 mM H_2O_2 and decanoic acid as substrate (as described in Table S8, entry 6) with dodecanoic acid (t_{ret} 10.3 min) as internal standard

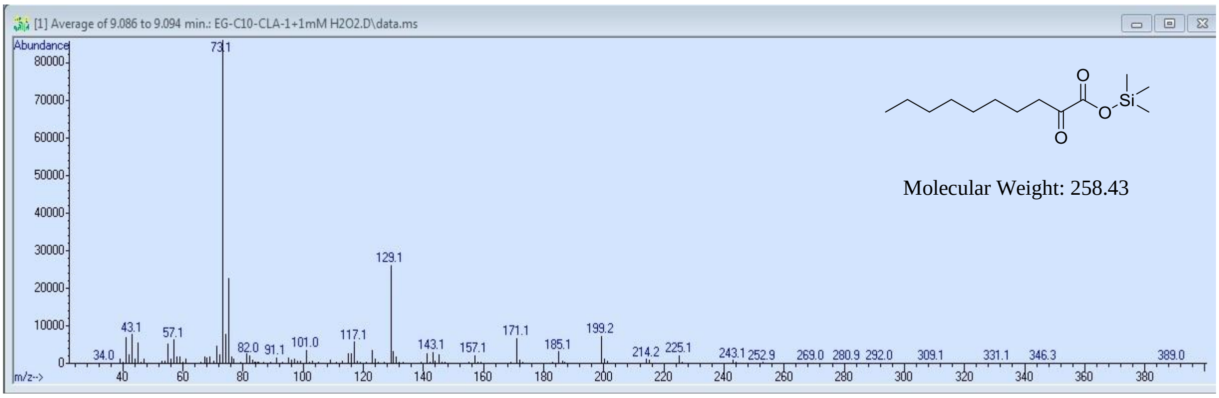

Figure S33. MS of peak of derivatized 2-oxodecanoic acid at 9.1 min from Figure S32

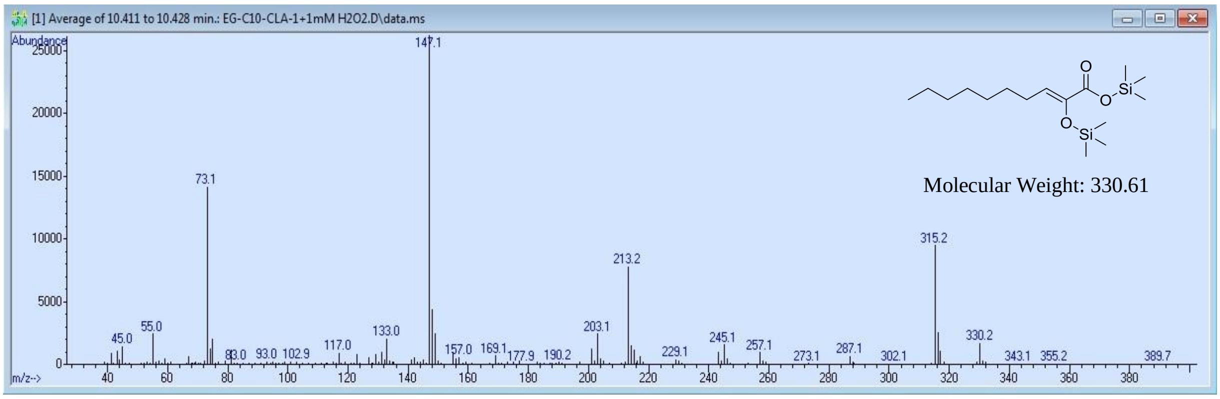

Figure S34. MS of peak of derivatized 2-oxodecanoic acid at 10.4 min from Figure S32

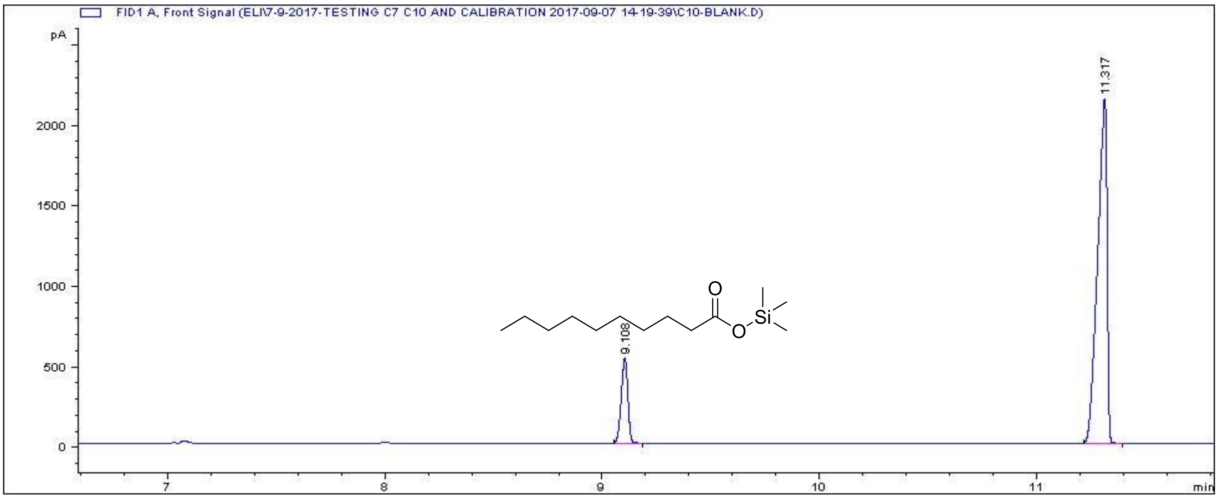

Figure S35. Achiral GC chromatogram of derivatized decanoic acid with dodecanoic acid (t_{ret} 11.3 min) as internal standard

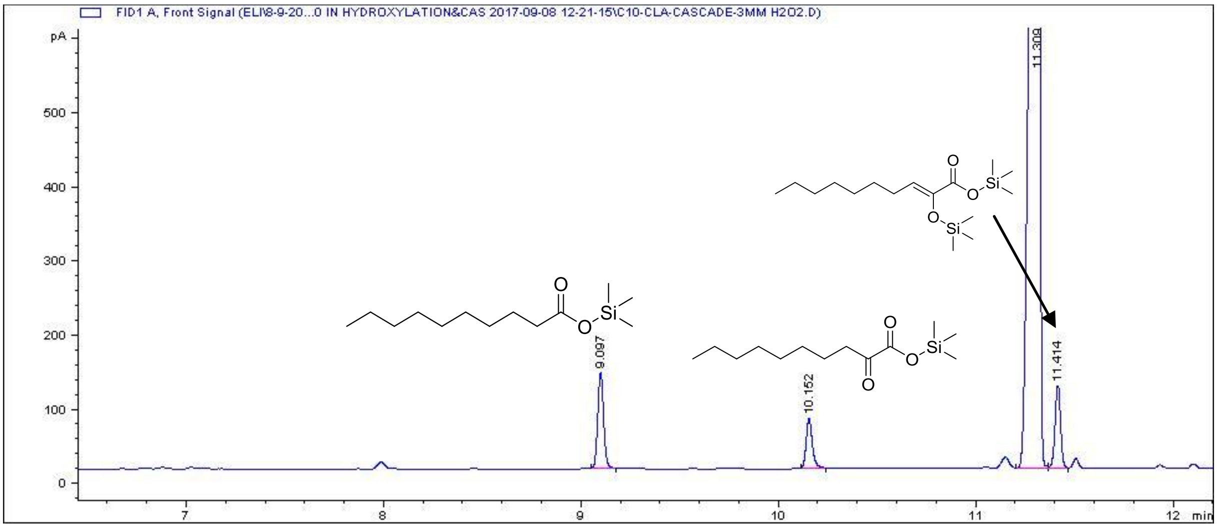

Figure S36. Achiral GC chromatogram obtained from cascade A using 3.0 mM H_2O_2 and decanoic acid as substrate (as described in Table S8, entry 6) with dodecanoic acid (t_{ret} 11.3 min) as internal standard

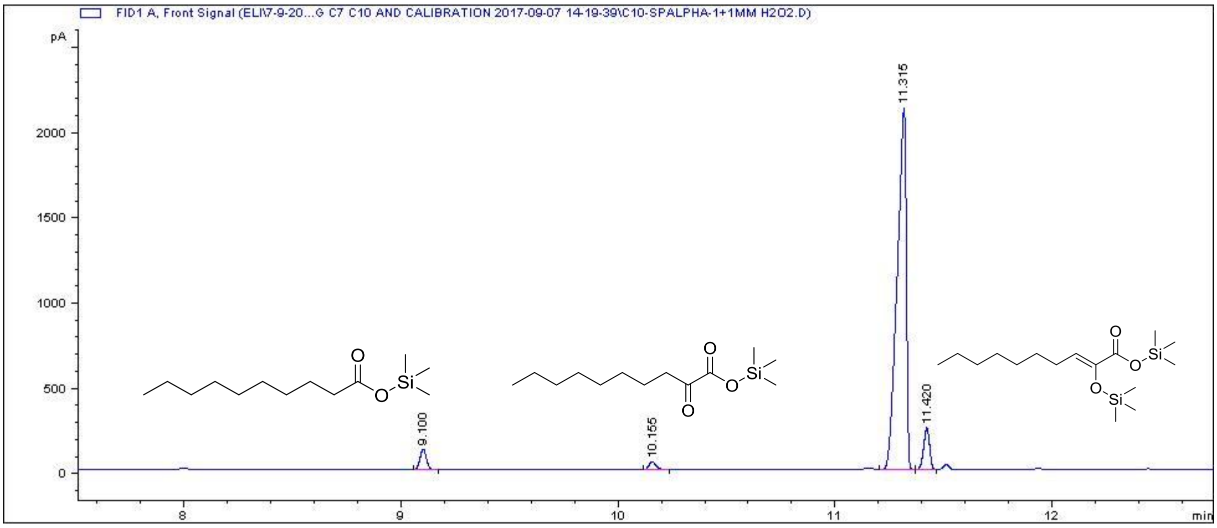

Figure S37. Achiral GC chromatogram obtained from cascade B using 1.0+1.0 mM H_2O_2 and decanoic acid as substrate (as described in Table S8, entry 5) with dodecanoic acid (t_{ret} 11.3 min) as internal standard

4. References

[1] a) J. A. Amaya, C. D. Rutland, T. M. Makris, *J. Inorg. Biochem*. **2016**, *158*, 11-16; b) C. Y. Seiler, J. G. Park, A. Sharma, P. Hunter, P. Surapaneni, C. Sedillo, J. Field, R. Algar, A. Price, J. Steel, A. Throop, M. Fiacco, J. LaBaer, *Nucleic Acids Res.* **2014**, *42*, D1253-D1260.

[2] M. Girhard, S. Schuster, M. Dietrich, P. Durre, V. B. Urlacher, *Biochem. Biophys. Res. Commun*. **2007**, *362*, 114-119.

[3] A. Dennig, M. Kuhn, S. Tassoti, A. Thiessenhusen, S. Gilch, T. Bülter, T. Haas, M. Hall, K. Faber, *Angew. Chem. Int. Ed.* **2015**, *54*, 8819-8822.

[4] J. Nazor, S. Dannenmann, R. O. Adjei, Y. B. Fordjour, I. T. Ghampson, M. Blanusa, D. Roccatano, U. Schwaneberg, *Protein Eng. Des. Sel*. **2008**, *21*, 29-35.

[5] a) T. Omura, R. Sato, *J. Biol. Chem*. **1964**, *239*, 2379-2385; b) T. Omura, R. Sato, *J. Biol. Chem.* **1964**, *239*, 2370-2378.

[6] B. B. Sheng, J. Xu, Y. S. Ge, S. Zhang, D. Q. Wang, C. Gao, C. Q. Ma, P. Xu, *ChemCatchem* **2016**, *8*, 2630-2633.

[7] K. Yorita, K. Aki, T. Ohkuma-Soyejima, T. Kokubo, H. Misaki, V. Massez, *J. Biol. Chem.* **1996**, *271*, 28300-28305.

[8] E. W. van Hellemond, M. van Dijk, D. P. H. M. Heuts, D. B. Janssen, M. W. Fraaije, *Appl. Microbiol. Biotechnol.* **2008**, *78*, 455-463.

[9] I. Matsunaga, M. Yamada, E. Kusunose, Y. Nishiuchi, I. Yano, K. Ichihara, *FEBS Lett.* **1996**, *386*, 252-254.

[10] A. Lopalco, G. Dalwadi, S. Niu. R. L. Schowen, J. Douglas, V. J. Stella. *J. Pharm. Sci.* **2016**, *105*, 705-713.
